# Novel Influences of IL-10 on CNS Inflammation Revealed by Integrated Analyses of Cytokine Networks and Microglial Morphology

**DOI:** 10.3389/fncel.2017.00233

**Published:** 2017-08-14

**Authors:** Warren D. Anderson, Andrew D. Greenhalgh, Aditya Takwale, Samuel David, Rajanikanth Vadigepalli

**Affiliations:** ^1^Daniel Baugh Institute for Functional Genomics/Computational Biology, Department of Pathology, Anatomy and Cell Biology, Thomas Jefferson University Philadelphia, PA, United States; ^2^Center for Research in Neuroscience, The Research Institute of the McGill University Health Center Montreal, QC, Canada

**Keywords:** CNS inflammation, microglia morphology, TNFα, IL-10, cytokine adaptation

## Abstract

Coordinated interactions between cytokine signaling and morphological dynamics of microglial cells regulate neuroinflammation in CNS injury and disease. We found that pro-inflammatory cytokine gene expression *in vivo* showed a pronounced recovery following systemic LPS. We performed a novel multivariate analysis of microglial morphology and identified changes in specific morphological properties of microglia that matched the expression dynamics of pro-inflammatory cytokine TNFα. The adaptive recovery kinetics of TNFα expression and microglial soma size showed comparable profiles and dependence on anti-inflammatory cytokine IL-10 expression. The recovery of cytokine variations and microglial morphology responses to inflammation were negatively regulated by IL-10. Our novel morphological analysis of microglia is able to detect subtle changes and can be used widely. We implemented *in silico* simulations of cytokine network dynamics which showed—counter-intuitively, but in line with our experimental observations—that negative feedback from IL-10 was sufficient to impede the adaptive recovery of TNFα-mediated inflammation. Our integrative approach is a powerful tool to study changes in specific components of microglial morphology for insights into their functional states, in relation to cytokine network dynamics, during CNS injury and disease.

## Introduction

The interplay between pro and anti-inflammatory cytokines in the central nervous system (CNS) determines the outcome of inflammation after CNS injury and in disease (DiSabato et al., 2016). Neuroinflammation is regulated by cytokines that interact through complex signaling networks (Benveniste, 1992; Codarri et al., 2010; Crotti and Ransohoff, 2016), and functions related to morphological properties of microglia (Kettenmann et al., 2013). While microglia-mediated neuroinflammation is a signature of infection, neurotrauma, and many neurological, neurodegenerative, and psychiatric diseases (David et al., 2015; Witcher et al., 2015; Hong et al., 2016; Vasek et al., 2016), microglia have homeostatic roles which are important for neural development and synaptic function (Tremblay et al., 2010; Schafer et al., 2012; Sipe et al., 2016). The complexity of the neuroinflammatory response is such that it can be both detrimental and beneficial to CNS injury and recovery, depending on the timing of inflammation and context of the injury (Sierra et al., 2013; David et al., 2015; Gadani et al., 2015). The pro-inflammatory cytokine TNFα and the anti-inflammatory cytokine interleukin-10 (IL-10) are known to play key roles in the balance of inflammation and are mediators in many CNS injuries and diseases (Ishii et al., 2013; Montgomery et al., 2013; Kroner et al., 2014; Chakrabarty et al., 2015; Guillot-Sestier et al., 2015; Madsen et al., 2016). However, a basic understanding of the kinetics of the TNFα response in relation to the anti-inflammatory control by IL-10 is lacking.

In our previous work, we performed an extensive literature search and formulated a microglia-specific cytokine network (Anderson et al., 2015). Based on this network, we developed a mathematical model of cytokine regulatory dynamics. Our microglia model predicted counter-intuitively that the anti-inflammatory cytokine intereukin-10 (IL-10) impedes adaptation or recovery of pro-inflammatory TNFα levels to baseline under conditions of inflammatory stimulation. This prediction was supported by *in vitro* evidence using bone marrow-derived macrophages (Anderson et al., 2015). However, this has yet to be validated in cultures of adult microglia or *in vivo*.

In addition to the production and response to cytokines, microglia exhibit a range of morphologies that reflect changes in function, their response to injury and disease, and their ability to shape the neuroinflammatory microenvironment (Walker et al., 2014). In response to injury, microglia alter their morphology within minutes (Davalos et al., 2005; Hines et al., 2009) and long-term changes in microglial morphology have been related to impairments in their immune function (Erny et al., 2015). Morphological changes in microglia are essential for processes such as phagocytosis (Tremblay et al., 2010; Abiega et al., 2016); however, it is not known if, and how, changes in cytokine networks during inflammation induce changes in microglial morphology.

In the present work, we investigated whether IL-10 controls TNFα expression dynamics and changes in microglial morphology in response to inflammation. To assess microglial morphology, we developed a novel unsupervised, multivariate analysis that is capable of detecting subtle changes in morphological parameters. We show here that IL-10-mediated feedback inhibition of TNFα *in vivo* after LPS stimulation influences adaptation of both TNFα expression and microglial morphology. We developed a novel mathematical model of multi-cellular cytokine networks *in vivo* which illuminated potential feedback interactions that could explain our CNS data in response to systemic LPS stimulation.

This combined analysis of cytokine networks and morphological changes in microglia could serve as a powerful approach to examine pathological CNS states and responses to interventions.

## Materials and methods

### Animals

All procedures were approved by the Animal Care Committee of the Research Institute of the McGill University Health Centre and followed the guidelines of the Canadian Council on Animal Care and the ARRIVE guidelines for reporting animal research (Kilkenny et al., 2010). Male C57BL/6 (WT) mice or *IL-10*^−/−^ mice on the same background (age 8–12 weeks) (Siqueira Mietto et al., 2015) were kept under a 12 h light/dark cycle with *ad libitum* access to food and water.

### Model of CNS inflammation

Mice were injected intraperitoneally with saline or Escherichia coli lipopolysaccharide (LPS; 0.33 mg/kg; serotype 0111:B4, Sigma; *n* = 3–4 per group) and sacrificed at 24 h, 3 and 5 days after injection.

### Cytokine profiling

We performed quantitative real time polymerase chain reactions (RT-qPCR) to assay for the expression levels of multiple cytokines. Mice were deeply anesthetized by intraperitoneal injection of ketamine (50 mg/kg), xylazine (5 mg/kg), and acepromazine (1 mg/kg), cardiac perfused (0.1 M PBS, pH 7.4) 6, 24 h, 3, or 5 days after injection, and brains or spinal cords snap-frozen for analysis. Tissue was homogenized and total RNA was extracted using the RNeasy Lipid Tissue Kit (Qiagen, CA). Reverse-transcription was performed with the Omniscript Reverse Transcription Kit (Qiagen, CA), and qPCR was performed using 1 mL of cDNA with Fast SYBR Green Master Mix (Applied Biosystems, CA) on a Step-One Plus qPCR machine (Applied Biosystems). Peptidylprolyl isomerase A (PPIA) was used as an internal control gene. The 2^−ΔΔCt^ method was used to calculate the cDNA expression fold change following standardization relative to PPIA (Schmittgen and Livak, 2008). All primers had a Tm of 60° C. Primer sequences were as follows: *Tnf* —fwd: 5′ TTG CTC TGT GAA GGG AAT GG 3′, rev 5′ GGC TCT GAG GAG TAG ACA ATA AAG 3′; *Il6—*fwd 5′ CTT CCA TCC AGT TGC CTT CT 3′, rev 5′ CTC CGA CTT GTG AAG TGG TAT AG 3′; *Il1b*—fwd 5′ ATG GGC AAC CAC TTA CCT ATT T 3′, rev 5′ GTT CTA GAG AGT GCT GCC TAA TG 3′; *Tgfb1*—fwd 5′ CTG AAC CAA GGA GAC GGA ATA C 3′, rev 5′ GGG CTG ATC CCG TTG ATT T 3′.

### Tissue processing and morphological image analysis

Animals were deeply anesthetized as mentioned above and perfused via the heart with 4% paraformaldehyde in 0.1 M PBS, pH 7.4. Thoracic spinal cords segments were removed and processed for cryostat sectioning (30 μm-thick coronal sections). Immunofluorescence was performed using rabbit anti-Iba1 (1:1,000; Wako) and detected using secondary antibody anti-rabbit Alexa Fluor 488 (1:500; Invitrogen). Sections were visualized using a confocal laser scanning microscope (FluoView FV1000; Olympus) and 30 μm z-stacks were prepared using FV10-ASW 3.0 software (Olympus). Iba1-positive microglia were imaged from the dorsal horn gray matter of the spinal cord. A total of 218 microglia from 28 mice were reconstructed with semi-automated procedures using IMARIS software (Oxford Instruments). Every complete microglia (full cell body and processes within the 30 μ m z-stack) within the field of view was reconstructed. The IMARIS software facilitates a semi-automated, interactive filament tracing method to reconstruct cells contained within confocal image stacks. Selected cells were subjected to the FilamentTracer algorithms that estimate the numeric values of features including geometric properties of the somata and processes. The FilamentTracer processed one channel (color) at a time, extracted objects corresponding to somata and process segments, and quantified lengths, areas, volumes, and between-segment angles. Following the automatic extraction of geometric features, manual editing was performed to delete erroneous process segments. Importantly, all analyses were completed in an unbiased manner, with respect to cell selection, by an individual blinded to the experimental conditions.

### Time-series analysis

Our general approach to statistically evaluating differences between the WT and KO conditions entailed comparing temporal profiles rather than individual data points (Storey et al., 2005; Anderson et al., 2017). We used kernel density plots visualize the distributions of morphological variables using the *beanplot* package in R (Kampstra, 2008). To determine whether particular morphological features exhibited differential dynamic profiles in WT versus IL-10 KO microglia, we implemented the optimal discovery procedure (Storey et al., 2007), as documented in our recent work (Anderson et al., 2017). According to this method, we fitted natural cubic splines to a given feature's temporal profile for each genotype and compared the computed error (i.e., sum of squared error, SS) to the error obtained if a single spline was fitted to the entire data set without regard for genotype. The latter error is termed *SS*^0^ and the former is *SS*^*A*^, corresponding to the null and alternative hypotheses, respectively. For a given feature *i*, a statistic was computed to describe the relative increase in goodness of fit achieved by including genotype-specific spline models: $F_{i}=\left(SS_{i}^{0}-SS_{i}^{A}\right)/SS_{i}^{A}$. The distribution of this statistic was estimated using bootstrap re-sampling and the resulting *p*-values were computed and corrected for multiple testing (Storey et al., 2005, 2007). This analysis was implemented using the *EDGE* package for the statistical programming language R (Storey et al., 2015; R-Core-Team, 2016). In particular, because we were comparing overall temporal profiles defined by spline fits, rather than individual data points, *post-hoc* analyses of genotype differences at specific time points were not applicable. Rather, our analyses provided information as to whether the general dynamic profiles differed as a function of genotype. For instance, consider the plot for soma area in **Figure 3C**. Our ODP analyses showed that fitting two spline curves, one for each genotype (black and magenta), resulted in a superior fit compared to fitting a single curve to the entire data set, based on the F statistic and associated corrected *p*-value (i.e., *q*-value) generated by our analysis (*q* = 0.04, see **Table 2**). This finding suggests that the dynamic responses of the soma area following an inflammatory trigger were distinct with respect to genotype.

### Principal component analysis (PCA)

PCA was utilized to illuminate inter-cellular relationships defined by multivariate measures that can be visualized in two or three dimensions. This is accomplished by projecting the multivariate data set onto a basis defined by coordinates aligned with vectors through highly variable regions of feature space. Importantly, groups of cells with distinguishable phenotypes can often be categorized by spatially separated projections in PC space. The respective projections of the cellular data in the principal component subspace are known as “scores” that are determined by “loadings” which indicate the relative contributions of each variable to the separation of cellular score data. Hence, variables or features with higher absolute loadings, corresponding to the PCs utilized for the data reduction, have a greater influence on the representation of the data in the PC space. Further details regarding the theoretical background and implementation details of our PC analysis are available in our previous work (Anderson et al., 2016).

### Feature selection

We identified features for NMF analysis as follows. We considered morphological features, or their distribution statistics, with ODP *P* < 0.05 or PCA loading magnitude > 0.2 (computed across the first four PCs (Anderson et al., 2016)) as the most significant features (*n* = 65) for further analysis (**Table 2**). Note that in some cases either the loadings or the ODP *p*-values did not meet our criteria. This “inclusive” feature selection approach allowed us to include features that showed substantial variation without significant differences in temporal profiles (based on α = 0.05), or significant genotype-specific temporal dynamics without substantial contributions to the variance (based on the loading magnitude cutoff of 0.2).

### Non-negative matrix factorization (NMF)

The NMF analysis incorporates elements of both dimensionality reduction and clustering to determine groups of features that are associated with varying degrees of expression in distinct clusters of cells (Brunet et al., 2004). Our NMF analysis was applied to decompose a data matrix **D**, with morphological features corresponding to rows and samples representing each column, into two non-negative matrices—the basis matrix **W** and the coefficient matrix **H**—such that the data matrix **D** is proportional to the product of the basis and coefficient matrices (**D** ≃ **WH**) (Lee and Seung, 1999). **W** provided basis vectors for the projection of coefficients **H** for each sample. The rows of **W** correspond to the features, and each column of **W** represents a meta-feature. Each meta-feature is a basis vector with a weight for each feature. Because NMF algorithms are generally designed to optimize sparsity of **W** and **H** (Gaujoux and Seoighe, 2010), most elements in each meta-feature are close to zero and all other elements compose a feature set that defines the meta-feature. The coefficient matrix **H** has a column for each sample and a row for each meta-feature. Hence, element (*i, j*) of **H** (i.e., *H*_*i,j*_) represents the contribution of meta-feature *i* to the morphological profile of sample *j*.

In implementing NMF, there are a number of ways to initialize **W,H** and to algorithmically refine the final matrices (Gaujoux and Seoighe, 2010). Further, the fidelity of the resulting factorization can be determined based on the likelihood that samples will cluster together across multiple iterations of the NMF algorithm, the degree of **W,H** sparsity, and/or the degree of agreement between the factorization product and the original data matrix (Brunet et al., 2004; Gao and Church, 2005; Cieślik and Bekiranov, 2014). We employed a plethora of methods to initialize the matrices (Brunet et al., 2004; Boutsidis and Gallopoulos, 2008; Marchini et al., 2013) and implement the refinement algorithm (Lee and Seung, 2001; Brunet et al., 2004; Badea, 2008; Gaujoux and Seoighe, 2010) and evaluated the results based on all aforementioned criteria. We found that singular value decomposition-based initialization (Boutsidis and Gallopoulos, 2008) coupled with optimization using a modified version of the original NMF algorithm (Lee and Seung, 1999), in which the objective function contains an offset term that accounts for features expressed at constant levels across samples (Badea, 2008), provided a viable approach when considering all fidelity criteria. Our implementation of NMF established a number of morphological cell states based on a predefined “rank” term. We implemented the NMF analysis with ranks spanning the range 1–12. We settled on rank = 6 as this setting facilitated interpretability of the resulting factorization at minimal expense of error and sparsity, consistent with previous approaches (Brunet et al., 2004).

The basic computations underlying NMF are illustrated in **Figure 4A**. In matrix computation, the element of the Data matrix in the top left corner (1,1) is computed by taking every element in the first row of **W**, multiplying each of these values by the corresponding values in first column of **H**, and taking the sum of these products. To compute the value of the Data matrix in the second row of the first column (2,1), take the sum of multiples of the second row of **W** and the first column of **H**. To compute the value in the second row of the second column (2,2), take the sum of multiples of the second row of **W** and the second column of **H**. Thus, the Data heatmap is organized based on the organization of both **W** and **H**.

### Multidemensional scaling (MDS)

MDS is a commonly employed method for dimensionality reduction (Park et al., 2014). This analysis was based on distances d_i,j_ computed using the Spearman rank correlation coefficients ρ_i,j_ between two cells i and j where the correlation was computed across all features: d_i,j_ = 1 − ρ_i,j_. The MDS algorithm was used to plot cellular data in 3D by minimizing the difference between Euclidean distance and distance in MDS space, where inter-cellular distance was defined by the correlation based distance metric d. In contrast to PCA, MDS facilitates the distinction of cell classes based on nonlinear relationships between features. We employed nonmetric MDS as described in detail previously (Park et al., 2014).

The MDS analysis was based on the correlation data from **Figure 5D** and provides a simplified representation of each sample as a point in 3D space such that the distances between points were scaled by the correlation between the respective samples. Samples with high correlations were represented by points that were close together. Samples with negative correlations are represented by points that were farther apart (**Figure 5E**). Both the correlation data and its transformation into MDS coordinates—when organized or colored based on the NFM clusters—showed that within a given NMF cluster, the global morphological profiles were similar. This analysis provides an independent way to view the similarities/differences within and between NMF-based clusters. The generally high correlations within clusters, and close cluster groupings in MDS-space, independently support the specificity of the NMF-based cluster/class identification.

### Morphological adaptation analysis

The adaptive recovery of individual morphological properties was assessed as follows. We first computed Z-score averages at each time point, and scaled these values to the interval $\overset{®}{Z}\in$ [0,1]. We implemented this scaling transformation because our main interest was in evaluating the relative extent of recovery to baseline following the peak response to LPS. Because some temporal profiles of morphological variables decreased (as opposed to increased) following LPS application, we evaluated whether adaptation should be considered based on recovery to baseline following an increase or decrease in each morphological feature (see **Figure 3C**). We evaluated the maximal deviation from baseline by computing the following quantities:

$$\begin{array}{l} \Delta max=|{\overset{®}{Z}}_{max}-{\overset{®}{Z}}_{t0}|\\ \Delta min=|{\overset{®}{Z}}_{min}-{\overset{®}{Z}}_{t0}| \end{array}$$

where ${\overset{®}{Z}}_{max}$ is the maximal averaged scaled Z-score, ${\overset{®}{Z}}_{min}$ is the minimal averaged scaled Z-score, ${\overset{®}{Z}}_{t0}$ is the averaged scaled Z-score under baseline conditions (t = 0 days), and **|.|** is the absolute value of the argument. For cases in which Δ*min* > Δ*max* was obtained, consistent with an LPS-mediated decrease in the corresponding morphological variable, we set the respective Z-score to $1-\overset{®}{Z}$ for our assessment of adaptation:

(1) $$\begin{array}{l} A=1-\frac{{\overset{®}{Z}}_{final}-{\overset{®}{Z}}_{t0}}{{\overset{®}{Z}}_{peak}-{\overset{®}{Z}}_{t0}} \end{array}$$

where ${\overset{®}{Z}}_{peak}$ is the mean peak Z-score and ${\overset{®}{Z}}_{final}$ is the mean Z-score at time *t* = 5 d. Note that we did not compare adaptation indices between WT and IL-10 KO in instances where one genotype showed an LPS-mediated increase whereas the other genotype showed an LPS-mediated decrease in a given morphological feature. The standard deviation of the adaptation metric *A* was estimated as follows using propagation of error (Anderson et al., 2015):

(2) $$\begin{array}{l} {\hat{\sigma }}_{A}^{2}={\sum_{\forall Z_{i}}SEM_{Z_{i}}^{2}\left(\frac{\partial A}{\partial {\bar{Z}}_{i}}\right)}^{2} \end{array}$$

where all $\forall {\overset{®}{Z}}_{i}$ refers to *Z*_*peak*_ and *Z*_*final*_, *SEM* refers to the estimated standard deviation of the mean ($standard\;deviation/\sqrt{n}$), and ${\overset{®}{Z}}_{0}$ was treated as a constant. Note that the *SEM* terms were computed using individual Z-score values transformed with the same scaling factors used to establish $\overset{®}{Z}\in$ [0,1], that is, the *min* and *max* terms applied for *Z*_*scaled*_ = (*Z* − *min*(*Z*)) / (*max*(*Z*) − *min*(*Z*)), as described above for the computation of *A*.

We compared adaptation between WT and IL-10 KO when the following conditions were met: *min*(*A*_*WT*_, *A*_*KO*_) > 0.5, *A*_*WT*_ > −0.2, *A*_*WT*_ < 1.2, *A*_*KO*_ > −0.2, and *A*_*KO*_ < 1.2. To evaluate the degree to which adaptation differed between WT and IL-10 KO, we estimated *p*-values as follows (Ogunnaike, 2011). We first defined the following T-statistic:

$$\begin{array}{l} T=\frac{A_{KO}-A_{WT}}{\sqrt{\frac{2S^{2}}{n_{min}}}},S=\frac{n_{min}-1}{df}\left({\hat{\sigma }}_{A,WT}^{2}+{\hat{\sigma }}_{A,KO}^{2}\right),\\ df=2n_{min}-2 \end{array}$$

where *n*_*min*_ is the minimal number of samples across all time points and both genotypes, considered for a given feature set. This choice of *n*_*min*_ tends to reduce the value of the T-statistic and therefore provides a more conservative estimate of its probability. We then used the t-distribution defined according to *df* degrees of freedom to estimate two-tailed *p*-values associated with *T* to test the null hypothesis that *A*_*KO*_ − *A*_*WT*_ = 0 against the alternative hypothesis that *A*_*KO*_ − *A*_*WT*_ ≠ 0. For each feature set, we adjusted the *p*-values for multiple testing by applying the Benjamini-Hochberg procedure with the *qvalue* package in R (Dabney et al., 2010). For our analyses of computed adaptation indices, we plotted errors associated with our adaptation indices based on estimates of 95 confidence intervals defined as follows:

$$\begin{array}{l} CI_{95}=A\pm t_{0.05/2}\left(df\right)\frac{{\hat{\sigma }}_{A}}{\sqrt{n_{min}}} \end{array}$$

### Statistical analysis

Statistical methods included the analysis of variance (ANOVA) with subsequent multiple comparison tests using the Fisher least significant difference (LSD) test with the Sidak correction (**Figures 6A,B**) and the hypergeometric test (using *phyper* in R). For the TNFα gene expression analysis depicted in **Figure 6A**, we performed a two-way ANOVA and then we compared the WT genotype to the IL-10 KO genotype at all time points. This analysis was motivated by our interest in investigating differences in the peak response between the WT and KO conditions. Similarly, for the analysis presented in **Figure 6B**, we utilized a two-way ANOVA. To specifically assess whether there were cases in which differences occurred relative to baseline for means computed across all features within given feature sets, we performed post hoc tests comparing feature set means to the mean at time = t0 within each genotype. In addition, we performed post hoc tests to compare the WT and IL10 KO means at each time point (see **Figure 6C**).

### Computational modeling

We developed a novel computational model to account for the dynamics of cytokine interactions between microglia and the CNS environment. Our model builds on a previous modeling formalism that we have successfully utilized to study cytokine interaction network dynamics in microglia *in vitro* (Anderson et al., 2015). The microglial compartment was formulated as follows to simulate cytokine expression dynamics:

(3) $$\begin{array}{l} \frac{dC_{x}}{dt}=\left(1+\frac{k_{x}\cdot LPS}{LPS+K_{LPS}}\right)\left(\prod_{i}\frac{C_{i}^{n_{ix}}}{C_{i}^{n_{ix}}+{K_{ix}}^{n_{ix}}}\right)\\ \;\left(\prod_{j}\frac{{K_{jx}}^{n_{jx}}}{C_{j}^{n_{jx}}+{K_{jx}}^{n_{jx}}}\right)-\gamma_{x}C_{x}-\gamma_{ss,x}C_{ss,x} \end{array}$$

(4) $$\begin{array}{l} C_{ss,x}=C_{x}\left(t=0\right)\\ \gamma_{ss,x}=\frac{\left(\prod_{i}\frac{C_{ss,i}^{n_{ix}}}{C_{ss,i}^{n_{ix}}+K_{ix}^{n_{ix}}}\right)\left(\prod_{j}\frac{K_{jx}^{n_{jx}}}{C_{ss,j}^{n_{jx}}+K_{jx}^{n_{jx}}}\right)-\gamma_{x}C_{x}}{C_{ss,x}} \end{array}$$

Cytokine expression *C*_*x*_ = *C*_*x*_(*t*) was modeled for the following cytokines: *x* = TNFα, IL-1β, IL-6, TGFβ, IL-10, and CCL5. Cytokine *x* could be produced at a maximal rate of 1+*k*_*x*_. The time-dependent production rate was modulated by activation from cytokines *C*_*i*_ and inhibition from cytokines *C*_*j*_. Activation and inhibition were modeled with sigmoidal functions characterized by half-maximal activation constant *K*_*ix*_ and cooperativity coefficient *n*_*ix*_. The degradation of *C*_*x*_ was modeled with both concentration-dependent and concentration-independent rate constants: γ_*x*_ and γ_*ss,x*_. The initial value of cytokine *x* was set to *C*_*ss,x*_ = 0.1 for all cytokines, and the concentration-independent degradation constant that was set to maintain a constant steady state (Equation 4) in the absence of stimulation. Based on available data, LPS stimulation was applied to all microglial species other than TGFβ as explained previously (Anderson et al., 2015). We set the stimulus duration to 16 h, but the qualitative characteristics of our simulation were robust to LPS stimulus duration.

We expanded our microglial model to incorporate the inflammatory influences of the CNS microenvironment. We formulated a CNS compartment of the model in which we assumed that astrocytes were the primary source of TGFβ–mediated feedback regulation of microglia (Norden et al., 2014). We simulated the delay between microglial IL-10 production and subsequent TGFβ production from the CNS environment (Norden et al., 2014) based on a series of coupled first-order systems (Ogunnaike and Ray, 1994):

(5) $$\begin{array}{l} \tau_{A}\frac{dA_{TGF}^{\left(1\right)}}{dt}=k_{A}\cdot M_{IL10}-\gamma_{A}\cdot A_{TGF}^{\left(1\right)} \end{array}$$

(6) $$\begin{array}{l} \tau_{A}\frac{dA_{TGF}^{\left(i\right)}}{dt}=k_{A}\cdot A_{TGF}^{\left(i-1\right)}-\gamma_{A}\cdot A_{TGF}^{\left(i\right)},i=1,2,\ldots ,n \end{array}$$

where *M*_*IL*10_ represents microglial IL-10, $A_{TGF}^{\left(i\right)}$ represents the *i*-th element in the cascade of terms leading up to CNS environment TGFβ $A_{TGF}^{\left(n\right)}$. The rate constants for the activation and degradation of the *A*_*TGF*_ terms are *k*_*A*_ and γ_*A*_, respectively, and τ_*A*_ is the time constant governing the interaction dynamics. This formulation has been utilized extensively in engineering applications involving time delays between the activation of process control components (Liu et al., 2013). We note that this representation of CNS neuroinflammation by a restricted repertoire of cytokines in only microglia and the CNS microenvironment is an oversimplification of the biological complexity. We address this issue in the discussion.

## Results

### *In vivo* analysis of cytokine expression and microglial morphology dynamics

**Figure 1:**
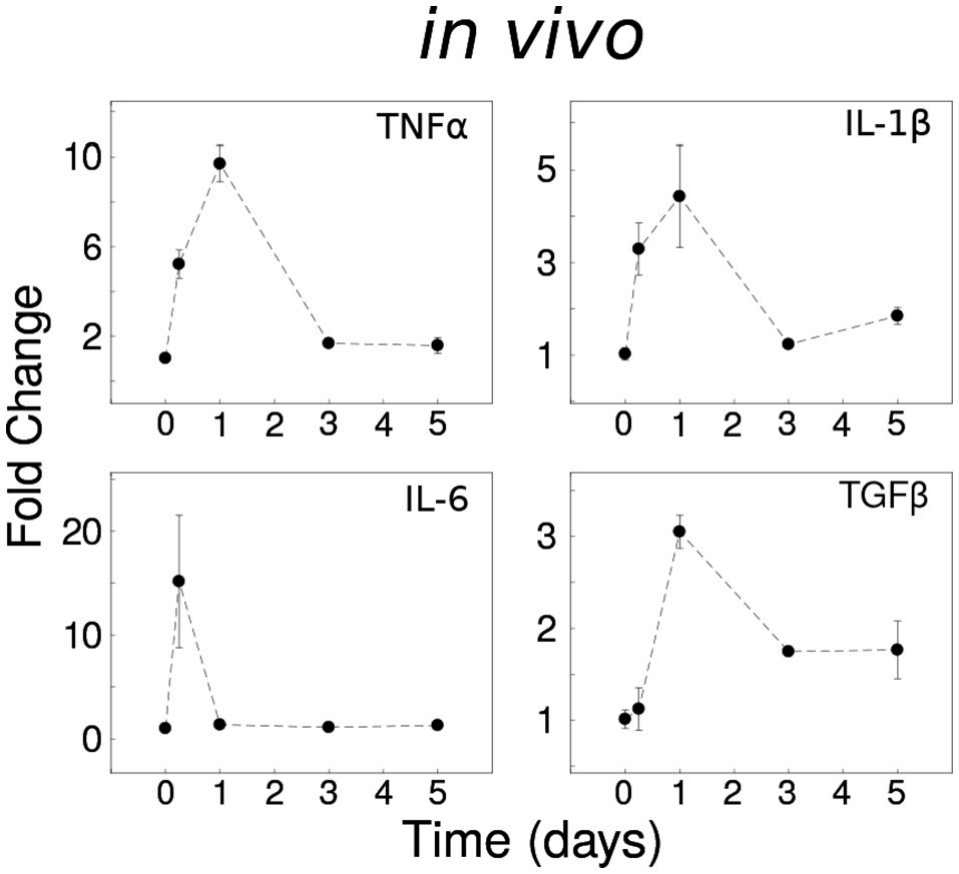
Dynamical analyses of cytokine network behavior *in vivo*. Spinal cord tissue cytokine gene expression data following systemic LPS (0.33 mg/kg i.p) at time = 0 (*n* = 3–4 mice per group).

To assess the *in vivo* expression of cytokines described as important by our previous *in silico* modeling, based on published data, we investigated the temporal responses to systemic LPS (0.33 mg/kg i.p) which elicits a pro-inflammatory cytokine response in the brain in adult mice (Henry et al., 2009; Fenn et al., 2012). The inflammatory responses of the CNS spinal cord *in vivo* were distinct from *in vitro* data (Anderson et al., 2015): (i) *Tnf* and *Il6* were rapidly expressed with fast recovery to the baseline level, (ii) *Il1b* showed rapid decay toward the baseline, and (iii) *Tgfb1* showed partial recovery of expression with sustained upregulation for 5 days (Figure 1). The prominent adaptive responses observed for *Tnf* and *Il6* suggest that these cytokines are regulated by robust negative feedback *in vivo*.

**Figure 2:**
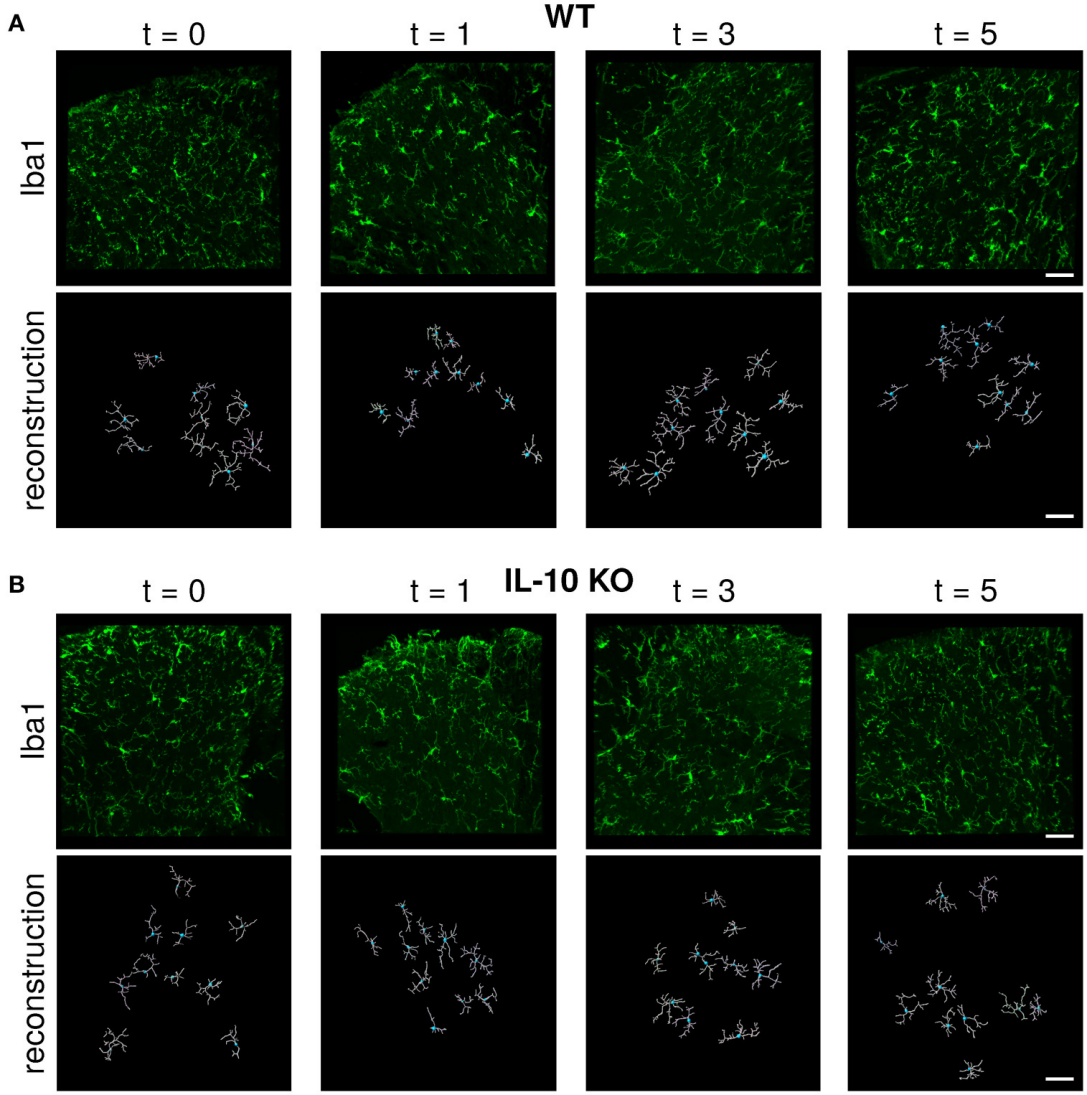
Immunofluorescent labeling and IMARIS reconstruction of microglial morphology in the spinal cord dorsal gray matter over time after LPS injection in WT and *IL-10* KO mice. Representative examples of Iba-1 labeled microglial morphology (green) in naive mice or 24 h, 3, and 5 days after systemic LPS (0.33 mg/kg i.p) in **(A)** WT and **(B)** IL-10^−/−^ mice. Below each Iba-1 image is the respective IMARIS reconstruction of microglial morphology (white). Dorsal horn gray matter was imaged, as shown. Scale bar = 50 μm.

**Figure 3:**
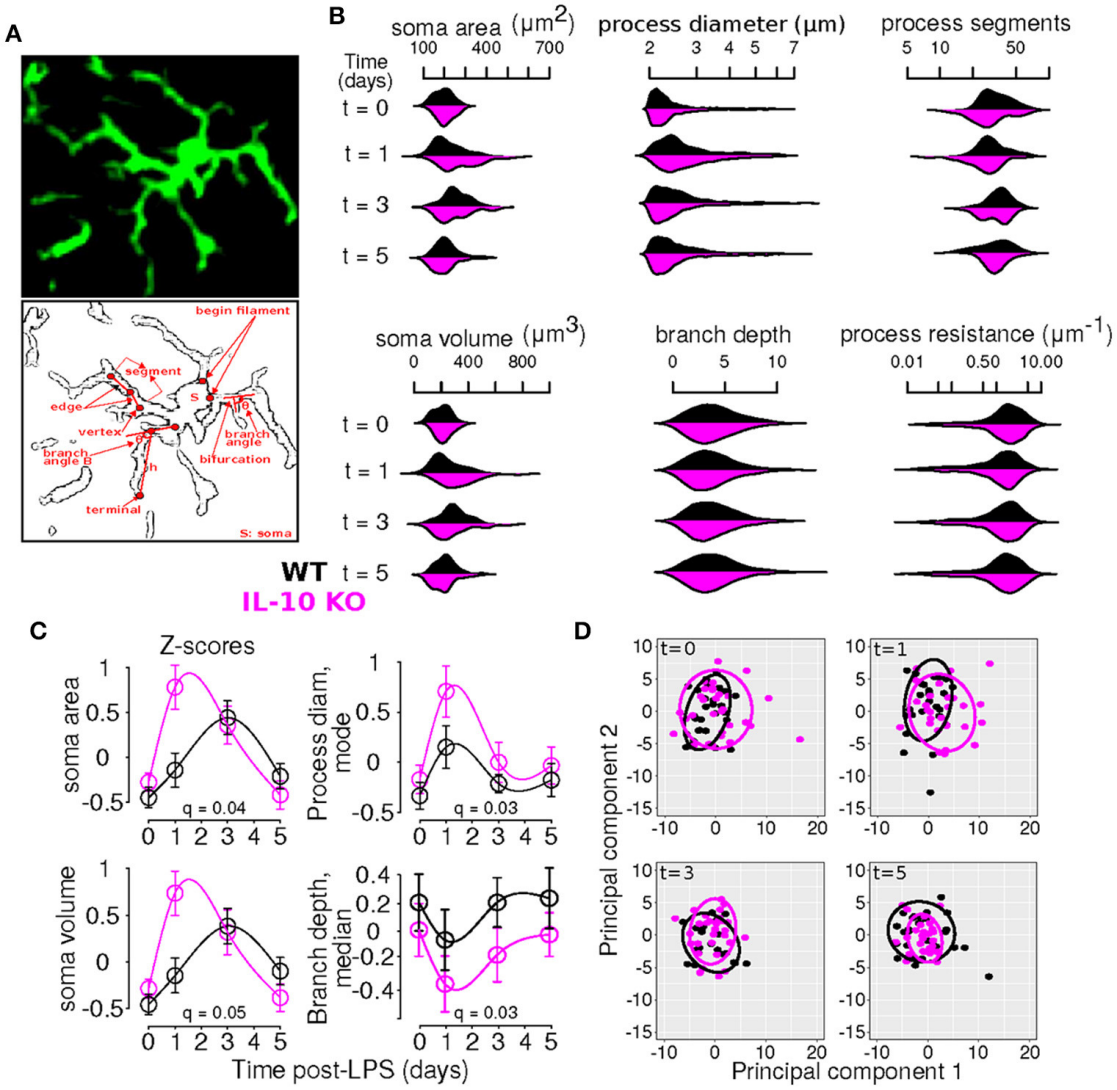
Time-series and multivariate statistical analyses reveal that IL-10 modulates the dynamics of microglial morphology. **(A)** Panels show an Iba1 stained microglia (top) and a line sketch showing some of the main morphological features analyzed (bottom). **(B)** Kernel density plots show the distributions of various morphological variables as a function of time for WT and *IL-10*^−/−^ (black and magenta, respectively). These analyses illustrate the relative influence of LPS on morphological properties in WT and *IL-10*^−/−^ microglia. **(C)** Morphological features with statistically different dynamic profiles between genotypes were analyzed using the optimal discovery procedure (ODP). Several features showed genotype-specific significant differences. As described in the methods, we used the ODP to compare the temporal profiles of WT and *IL-10*^−/−^ microglial features and we indicated the false discovery rate adjusted *p*-values (i.e., *q*-values) in the four panels; see Table 2 for documentation of all q-values. **(D)** Principal component analysis of differences in morphological features at various times after LPS administration. Note the difference between the two groups at *t* = 1 day.

Microglial morphology is critical for both the chemical and physical functions of microglia (Kettenmann et al., 2013; Morrison and Filosa, 2013; Šišková and Tremblay, 2013; Yamada and Jinno, 2013; Erny et al., 2015; Schafer and Stevens, 2015). Because previous studies have not compared the dynamic profiles of cytokine expression with the temporal responses of microglial morphology, we assessed microglial morphology over time following systemic LPS (Figure 2A). Based on our previous work, we hypothesized that IL-10 could regulate neuroinflammation through negative feedback, and we examined both wildtype (WT) and IL-10 knockout mice (*IL-10*^−/−^; Figure 2B). We reconstructed the morphological properties of 218 spinal cord microglia from WT and *IL-10*^−/−^. We analyzed an expansive set of geometrical features related to microglial soma and processes (Figure 3A, Table 1). Kernel density plots show the distributions of morphological variables as a function of time for WT and *IL-10*^−/−^ (black and magenta, respectively) (Figure 3B). These analyses illustrate the relative influence of LPS on morphological properties in WT and *IL-10*^−/−^ microglia over time. Note the non-Gaussian forms of the distributions, many of which exhibit long tails. In many cases, LPS (dosed IP at time = 0, t0) resulted in an apparent expansion of the distributions. These complexities highlight the utility of sensitive analytic methods for deciphering the temporal properties of microglial responses to neuroinflammation.

**Table 1:** IMARIS labels and descriptors.

**IMARIS label—features**	**Renamed label—features**	**Compartment**	**Units**	**Distributed**	**Feature set**	**Primary cluster**	**Description**
Area	Soma area	Soma	um^2^	No	fs2	c2	Surface area of the soma
Ellipsoid.Axis.Length.A	Soma ellipsoid length A	Soma	um	No	fs2	c2	Refers to parameters associated with ellipsoidal fits to somata
Ellipsoid.Axis.Length.B	Soma ellipsoid length B	Soma	um	No	fs2	c2	Refers to parameters associated with ellipsoidal fits to somata
Ellipsoid.Axis.Length.C	Soma ellipsoid length C	Soma	um	No	fs2	c2	Refers to parameters associated with ellipsoidal fits to somata
Ellipticity.oblate.	Ellipticity oblate	Soma	NA	No	NA	NA	Shape parameter of the soma
Ellipticity.prolate.	Ellipticity prolate	Soma	NA	No	fs2	c2	Shape parameter of the soma
Number.of.Triangles	Number triangles	Soma	Count	No	fs2	c2	Number of triangles, associated with resolution required for analysis
Number.of.Vertices	Number vertices	Soma	Count	No	fs2	c2	Number of vertices in a filament (a filament is a process emanating from the soma, along with all associated branches)
Number.of.Voxels	Number voxels	Soma	Count	No	fs2	c2	Number of voxels in the contour surface of the soma
Sphericity	Soma sphericity	Soma	NA	No	NA	NA	Shape parameter of the soma
Volume	Soma volume	Soma	um^3^	No	fs2	c2	Volume of the soma
Filament.No.Dendrite.Branc.28	Process branches	Process	Count	No	fs1	c1	The number of process branch points
Filament.No.Dendrite.Segments	Process segments	Process	Count	No	fs1	c1	Number of process segments in a filament
Filament.No.Dendrite.Termi.31	Process terminals	Process	Count	No	fs1	c1	Number of process terminals in a filament
Filament.No.Edges	Filament edges	Process	Count	No	fs1	c1	Number of connections between vertices in a filament
Filament.Volume.sum.	Filament volume	Process	um^3^	No	fs1	c1	Total volume of all processes of the cell
Dendrite.Area	Process area	Process	um^2^	Yes	fs4	c4-6	Surface area of a process segment (sum of areas encompassing edges)
Dendrite.Branch.Depth	Branch depth	Process	Count	Yes	fs1	c1	Number of process bifurcations along the shortest path from the soma to a given coordinate
Dendrite.Branch.Level	Branch level	Process	NA	Yes	fs1	c1	Metric of incremental decrease in dendrite diameter at each bifurcation point
Dendrite.Branching.Angle	Branch angle	Process	Degrees	Yes	fs3	c3	Angle between branches at a bifurcation point
Dendrite.Branching.Angle.B	Branch angle B	Process	Degrees	Yes	fs3	c3	Angle between the line from the beginning of a filament to a bifurcation and between a bifurcation and terminal
Dendrite.Length	Process length	Process	um	Yes	fs4	c4-6	Sum of edge lengths between bifurcation points
Dendrite.Mean.Diameter	Process diameter	Process	um	Yes	fs3	c3	Diameter of a process
Dendrite.Orientation.Angle	Process orientation angle	Process	Degrees	Yes	fs3,4	c3-6	Angle between image plane and line connecting the start to end of a branch
Dendrite.Position	Process position	Process	um	Yes	fs1,4	c1,4-6	Positional coordinate of a process
Dendrite.Resistance	Process resistance	Process	um^−1^	Yes	fs4	c4-6	Proxy for electrical length based on process length and cross-sectional area
Dendrite.Straightness	Process straightness	Process	NA	Yes	fs3,4	c3-6	Sum of edge lengths divided by distance from start to end of a segment (h)
Dendrite.Volume	Process volume	Process	um^3^	Yes	fs4	c4-6	Total filament volume including all segments

### Unsupervised, multivariate analysis of microglial morphology reveals distinct cell states characterized by functionally defined sets of morphological properties

To comprehensively analyze the features of microglia, such as number of branch points and branch point angles, which are distributed variables in single cells, we assessed distributional statistics related to center (mean, median, mode), spread (standard deviation, variance, coefficient of variation), and shape (skewness, and kurtosis), yielding 110 total features (Table 2). To elucidate the morphological features with statistically distinguishable dynamic profiles between WT and *IL-10*^−/−^, we employed the optimal discovery procedure (ODP) (Storey et al., 2005, 2007). This analysis facilitated the direct comparison of temporal profiles (smooth curves in Figure 3C), as opposed to individual data points, which is optimal for time-series analysis (Storey et al., 2005). Several features showed genotype-specific significant differences in the respective temporal dynamics (*P* < 0.05; Figure 3C; Table 2–*q*-values). For distributed features of microglial processes, we directly compared the underlying distributions using the Kolmogorov-Smirnov (K-S) test and observed several time- and genotype-specific differences (data not shown). To determine whether the temporal dynamics of global cell state variations were sensitive to IL-10, we applied principal components analysis (PCA) to our multivariate morphology data. This analyses revealed subtle but distinct quantitative morphological differences between WT and *IL-10*^−/−^ microglia, particularly in the initial phase of the response to LPS (*t* = 1 day; Figure 3D). Furthermore, our PCA data facilitated the identification morphological features with high variability that we utilized in downstream analysis (Table 2, see Methods–Feature selection).

**Table 2:** Genotype-specific differences in the temporal dynamics of features.

**Feature**	**Compartment**	**Feature set**	**Dist property**	**Loading**	***q*-value**
Process branches	Process	fs1	NA	0.24	0.023
Process segments	Process	fs1	NA	0.238	0.023
Process terminals	Process	fs1	NA	0.233	0.023
Filament edges	Process	fs1	NA	0.237	0.04
Filament volume	Process	fs1	NA	0.229	0.042
Branch depth_mean	Process	fs1	Center	0.213	0.028
Branch depth_median	Process	fs1	Center	0.203	0.03
Branch level_mean	Process	fs1	Center	0.189	0.015
Branch level_median	Process	fs1	Center	0.178	0.015
Branch level_mode	Process	fs1	Center	0.175	0.015
Process position_sdev	Process	fs1	Spread	0.206	0.078
Branch depth_sdev	Process	fs1	Spread	0.179	0.03
Soma area	Soma	fs2	NA	0.286	0.04
Soma ellipsoid length A	Soma	fs2	NA	0.216	0.113
Soma ellipsoid length C	Soma	fs2	NA	0.243	0.015
Number triangles	Soma	fs2	NA	0.29	0.041
Number vertices	Soma	fs2	NA	0.29	0.041
Number voxels	Soma	fs2	NA	0.288	0.052
Soma volume	Soma	fs2	NA	0.288	0.054
Ellipticity prolate	Soma	fs2	NA	0.136	0.04
Process diameter_mean	Process	fs3	Center	0.258	0.078
Process diameter_median	Process	fs3	Center	0.225	0.042
Process straightness_mean	Process	fs3	Center	0.205	0.113
Branch angle_mean	Process	fs3	Center	0.12	0.03
Process diameter_mode	Process	fs3	Center	0.139	0.03
Branch angle_95ci	Process	fs3	Spread	0.232	0.106
Branch angle B_95ci	Process	fs3	Spread	0.238	0.09
Process diameter_95ci	Process	fs3	Spread	0.226	0.148
Branch angle B_sdev	Process	fs3	Spread	0.197	0.04
Process orientation angle_95ci	Process	fs3	Spread	0.188	0.03
Branch angle B_skew	Process	fs3	Shape	0.248	0.03
Branch angle B_kurt	Process	fs3	Shape	0.217	0.099
Process area_mean	Process	fs4	Center	0.239	0.033
Process area_median	Process	fs4	Center	0.236	0.04
Process area_sdev	Process	fs4	Center	0.251	0.063
Process length_mean	Process	fs4	Center	0.245	0.04
Process length_median	Process	fs4	Center	0.221	0.066
Process resistance_mean	Process	fs4	Center	0.246	0.077
Process resistance_median	Process	fs4	Center	0.212	0.075
Process volume_mean	Process	fs4	Center	0.238	0.03
Process volume_median	Process	fs4	Center	0.238	0.03
Process area_mode	Process	fs4	Center	0.159	0.03
Process volume_mode	Process	fs4	Center	0.173	0.03
Process area_95ci	Process	fs4	Spread	0.242	0.023
Process area_cv	Process	fs4	Spread	0.283	0.125
Process length_sdev	Process	fs4	Spread	0.259	0.091
Process length_95ci	Process	fs4	Spread	0.25	0.03
Process length_cv	Process	fs4	Spread	0.281	0.115
Process resistance_sdev	Process	fs4	Spread	0.239	0.13
Process resistance_95ci	Process	fs4	Spread	0.245	0.042
Process resistance_cv	Process	fs4	Spread	0.245	0.1
Process volume_sdev	Process	fs4	Spread	0.228	0.04
Process volume_95ci	Process	fs4	Spread	0.228	0.015
Process volume_cv	Process	fs4	Spread	0.245	0.11
Process position_95ci	Process	fs4	Spread	0.174	0.015
Process area_skew	Process	fs4	Shape	0.27	0.115
Process area_kurt	Process	fs4	Shape	0.241	0.09
Process length_skew	Process	fs4	Shape	0.271	0.13
Process length_kurt	Process	fs4	Shape	0.239	0.106
Process resistance_skew	Process	fs4	Shape	0.238	0.13
Process resistance_kurt	Process	fs4	Shape	0.201	0.078
Process volume_skew	Process	fs4	Shape	0.205	0.109
Process orientation angle_kurt	Process	fs4	Shape	0.09	0.04
Process straightness_skew	Process	fs4	Shape	0.056	0.023

**Figure 4:**
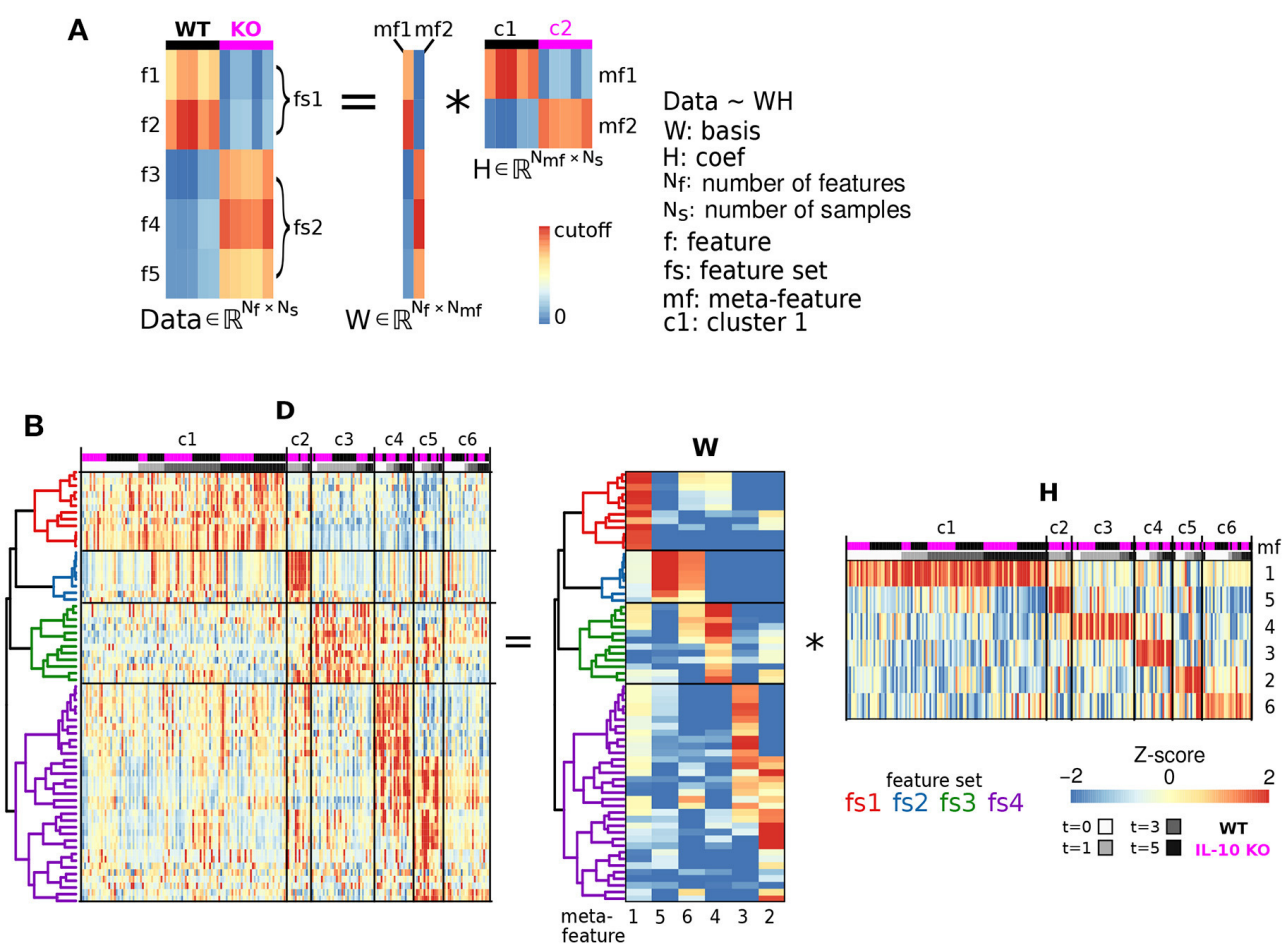
Morphological cell states characterized by distinct sets of morphological features. **(A)** Illustrative example of Non-negative matrix factorization (NMF) to show representations of a Data matrix, Basis matrix, and Coefficient matrix. **(B)** Microglial morphology data set organized according to feature sets. The Z-score matrix of morphological data **D** is organized based on the NMF analysis. The Basis matrix **W** of morphological meta-features is defined by four sets of features (fs1-4) from 218 reconstructed microglia, and the Coefficient matrix **H** shows a representation of meta-features in clusters of microglia (c1-6). ^*^Denotes multiplication.

We next examined whether genotype-specific microglial cell states could be segregated into distinct classes based on specific groups of morphological features. We employed non-negative matrix factorization (NMF), a highly effective technique to define groups of features that are associated with varying degrees of representation in distinct clusters of cells (Lee and Seung, 1999), which involves factorization of a data matrix **D** (Nf morphological features measured in Ns cells) into a basis matrix **W** of meta-features (a combination of individual features), and a coefficient matrix **H** with entries corresponding to the non-negative weight of each meta-feature in each sample (Figure 4A). We utilized NMF to test whether particular combinations of morphological features were overrepresented in specific subsets of the microglial population. Because the interpretability of multivariate analysis results is sensitive to the information content of the variables included in the analysis, we implemented a novel step-wise approach in which we initially identified informative features based on independent ODP and PC analyses. Figure 4B shows Z-score feature data with microglial cells (columns of the heatmap) organized based on the clustering of the coefficient matrix **H**, and rows organized based on the clustering of the basis matrix **W**. The clarity of the Z-score groupings, as indicated by the lines and diagonal pattern of ‘reddish blocks’, suggest that the **WH** decomposition (an estimate of two matrices whose product approximates but is not identical to the data set) provides a useful representation of the data that facilitates its interpretation. Thus, the Z-score data matrix (**D**) can be approximated by multiplying **W** and **H** (Figure 4B). This analysis identified four clusters of features (“feature sets,” fs1-4) and six clusters of discrete microglial cell states, each characterized by a specific profile of feature-set representation (c1-6; Figure 4B).

**Figure 5:**
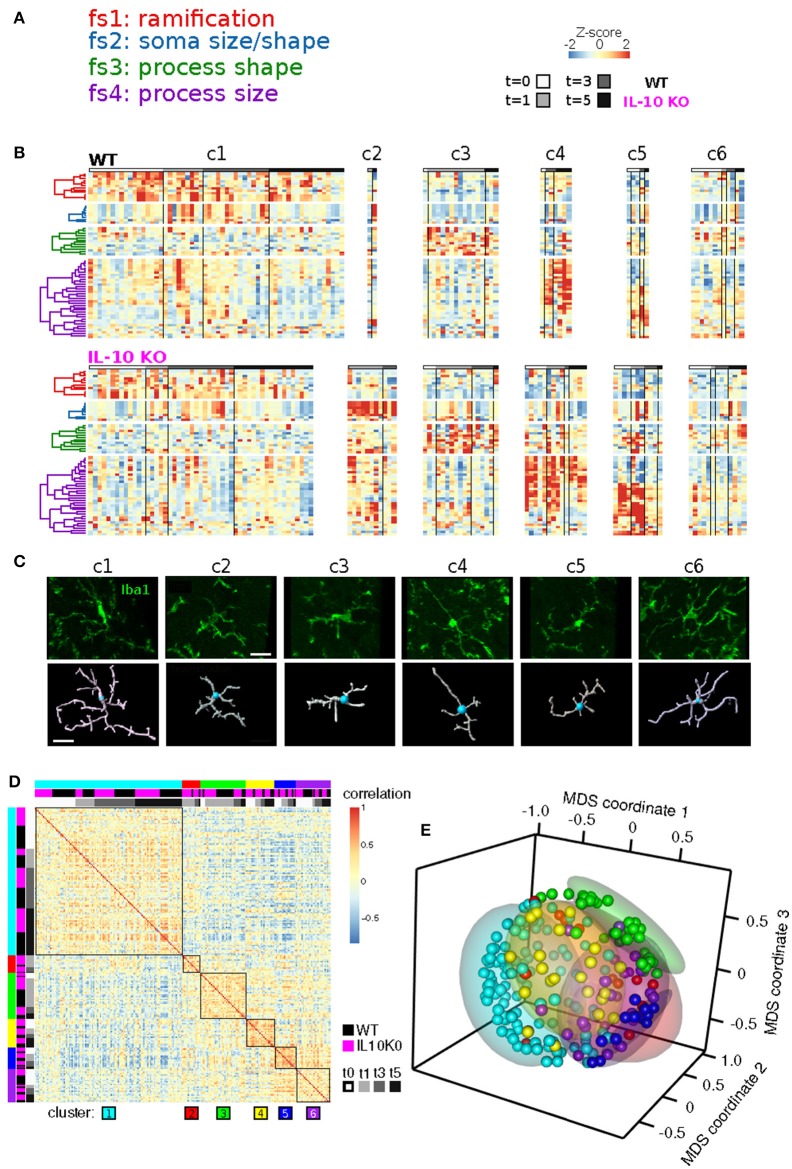
Temporal analysis of IL-10 influences on feature set dynamics. **(A)** List of morphological signatures of the 4 feature sets (see Table 2): ramification, soma size/shape, process shape, and process size. **(B)** Morphological cell state clusters (c1-6) are displayed for WT (top) and *IL-10*^−/−^ microglia (bottom). Note the 6 classes of microglia based on differences in feature set distribution, and differences in the population size of the different classes (c1-c6) between genotypes. **(C)** Representative confocal images of the six classes of cells and their confocal image-based reconstructions. Scale bar = 20 μm. **(D)** The Spearman rank correlation matrix shows that samples are highly correlated within the six clusters defined by NMF. **(E)** The Multidimensional scaling (MDS) analysis shows that samples of NMF-defined clusters are grouped together in a 3-dimensional projection according to MDS. These results independently support the findings from the NMF analysis.

Our analysis revealed four clusters of morphological features involving process ramification (fs1), soma size/shape (fs2), process shape (fs3), and process size (fs4) (Figure 5A; Table 2). There was considerable agreement between the features included in each feature-set and previously identified feature relationships (Yamada and Jinno, 2013; Kongsui et al., 2014). Feature set 1 (fs1) was associated with large branch arbors with numerous branch bifurcations and progressive decrements in thickness from soma to terminal (Table 2). This feature set was highly represented in both WT and *IL-10*^−/−^ microglia from cluster 1 (Figure 5B), with features indicative of the ramified morphology of healthy microglia. Fs2 was associated with larger soma size, consistent with microglial activation following LPS application (Kozlowski and Weimer, 2012). Interestingly, cluster 2 microglia with relatively high fs2 levels were observed primarily in *IL-10*^−/−^ (*P* = 0.019, hypergeometric test), indicating that this cell state may be associated with pro-inflammatory up-regulation associated with the absence of IL-10. Fs3 was associated with the complexity of branch shape, including features related to the angularity of branching, the functional significance of which is not clear (Karperien et al., 2013) and was represented equally in cluster 3 WT and *IL-10*^−/−^ microglia. Fs4 was associated with the size of the branches, indicated by features such as branch length. Cluster 4-5 microglia showed relatively high levels of fs4 morphological features and a trend toward predominant representation in *IL-10*^−/−^ microglia (*P* = 0.15). Cluster 6 microglia were characterized by moderate expression of all feature sets. Overall, the morphological feature sets could be summarized respectively to indicate the extent of ramification, soma size/shape, process shape, and process size (Figure 5A, Table 2).

When sample images of reconstructed microglia representative of each NMF-based cluster were examined, the six clusters were not easily or unambiguously identifiable by visual inspection (Figure 5C). The heterogeneity seen within and across microglial clusters highlights the need for detailed morphological analyses across a range of experimental contexts, based on large sample sizes of microglia, in an unbiased manner (Walker et al., 2014). To confirm that our NMF results could not be attributed to a spurious product of algorithmic processing, we implemented an independent analysis using sample-by-sample correlations and multidimensional scaling (Figures 5D,E; also see Methods). We found that clusters of samples, based on the NMF analysis, exhibited high correlations and relative proximity within MDS coordinates. Overall, our analyses revealed subtle differences in microglial morphology mediated by IL-10 in this model of CNS inflammation.

### IL-10 impedes adaptation of TNFα and microglial morphology

**Figure 6:**
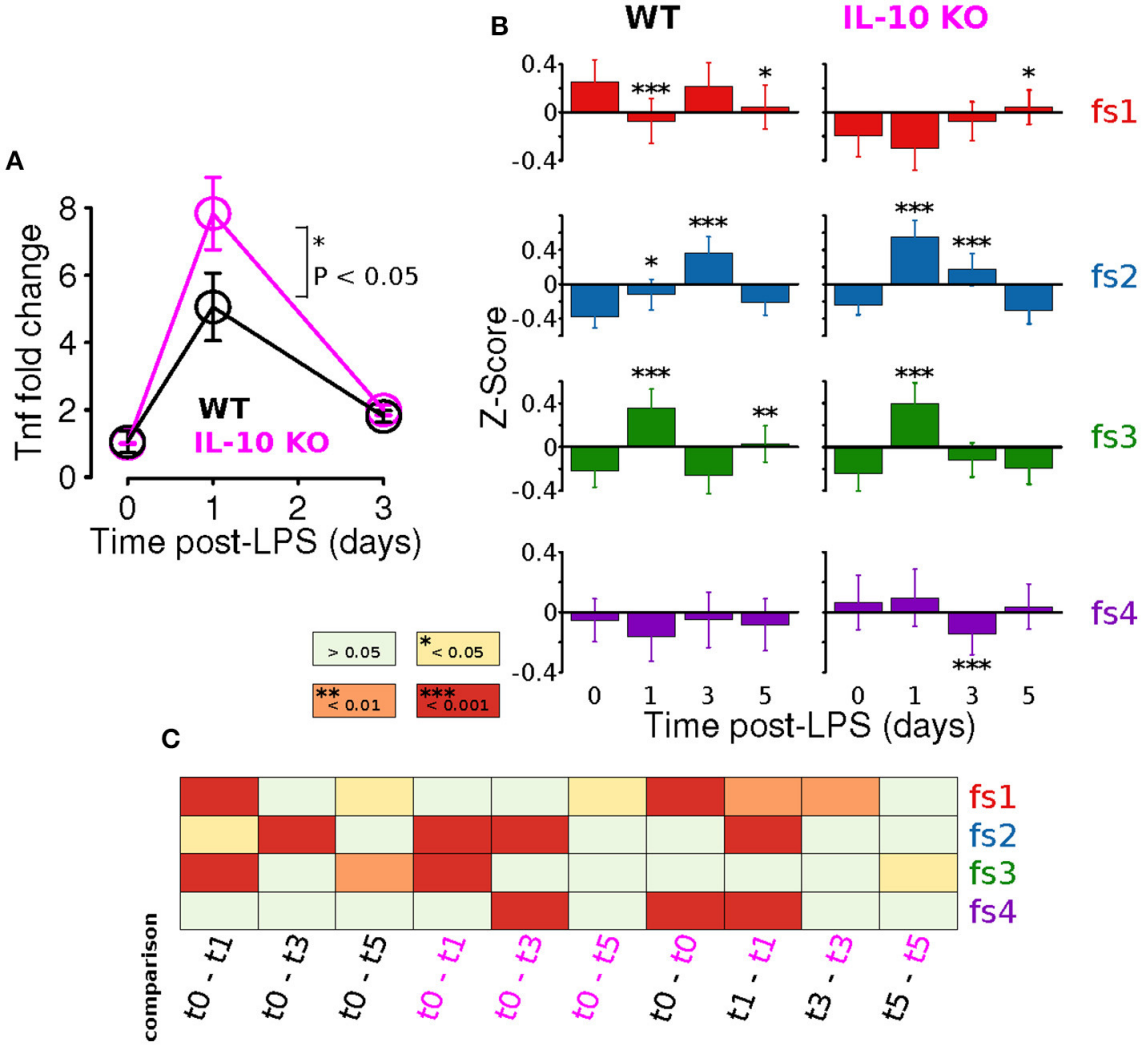
IL-10 restrains TNFα adaptation and regulates specific morphological features. **(A)** *In vivo* CNS gene expression of TNFα in WT and *IL-10*^−/−^ LPS-treated mice show that the absence of IL-10 is associated with an enhanced LPS response and corresponding enhanced adaptive recovery to baseline (two-way ANOVA with with Sidak's multiple comparisons *P* = 0.0147, *n* = 4 mice per group, brain tissue). **(B)** For both WT and *IL-10*^−/−^, the arithmetic means across each feature set (fs1-4) was computed at the four time points (*t* = 0, 1, 3, and 5 days). Temporal dynamics of each feature set was analyzed using a two-way ANOVA and *p*-values based on a Fisher LSD post-hoc test with a Sidak correction. **(C)** The heatmap depicts corrected *p*-values for a focused set of post hoc comparisons (columns) applied for each feature set (rows). For instance, the first column shows results for the comparison of the WT means at time 0 and time 1 day, the third column shows the comparison of the *IL-10*^−/−^ means at time 0 and time 1 day, and the last column shows the comparison of the WT and *IL-10*^−/−^ means at time 5 days.

Adaptation is an important emergent property of engineered feedback control systems and components of the immune system operating inside and out of the CNS (Brudecki et al., 2013; Anderson et al., 2015; Montefusco et al., 2016). Our previous study showed that IL-10 restrained the adaptation of the TNFα response to LPS *in vitro*. We directly tested the hypothesis that IL-10 represses adaptation of CNS TNFα expression in response to systemic LPS *in vivo* at peak (24 h) and recovery (3 days) of expression. We assessed the TNFα response to LPS (i.p.) in brain tissue. Despite reported microglial heterogeneity in the healthy CNS (Grabert et al., 2016), or during plasticity, we found no difference in TNFα expression the brain and spinal cord 24 h after LPS injection (Supplementary Figure 1). Our experimental results with *IL-10*^−/−^ mice showed, as predicted, a larger fold change in TNFα gene expression in the CNS following LPS (*P* < 0.05, two-way ANOVA with with Sidak's multiple comparisons *P* = 0.0147, Figure 6A). However, TNFα expression showed complete recovery to WT levels 3 days after LPS injection (Figure 6A). Thus, the absence of IL-10 resulted in enhanced adaptive recovery of the TNFα inflammatory response to LPS *in vivo*. These results suggest a novel hypothesis that the anti-inflammatory cytokine IL-10 restrains the degree of adaptive recovery following *in vivo* inflammation in the CNS.

We next assessed whether *IL-10*^−/−^ influences the adaptation of morphological properties in response to LPS stimulation, as was observed for TNFα gene expression *in vivo* (Figure 6A). For this analysis, the arithmetic mean levels of each feature-set were evaluated at each time-point. For all feature sets, we observed significant main effects for time [*P* < 0.001; *F*_(3, 2608)_ = 9.5, *F*_(3, 1736)_ = 56.1, *F*_(3, 2608)_ = 63.8, and *F*_(3, 5878)_ = 5.6 for fs1-4, respectively], and we observed significant main effects of genotype (*P* < 0.001) for every feature set other than fs3 [*P* = 0.85; *F*_(1, 2608)_ = 48.1, *F*_(1, 1736)_ = 18.0, *F*_(1, 2608)_ = 0.04, and *F*_(1, 5878)_ = 24.0]. Likewise, significant time-genotype interaction terms were observed for all feature sets (*F* > 5, *P* < 0.001), thus indicating that the occlusion of *Il10* modifies the temporal dynamics of the morphological response the systemic inflammation, consistent with our time series analysis. When the temporal dynamics of feature set averages were compared across genotypes, the mean response profiles for fs2 and fs3 appeared to be consistent with enhanced morphological adaptation in *IL-10*^−/−^, as compared to WT (Figure 6B). It is noteworthy that WT microglia showed peak fs2 responses at t = 3 d whereas *IL-10*^−/−^ microglial expression of fs2 features were maximal at t = 1 d following LPS. In contrast, the fs3 peak responses of both genotypes were observed at t = 1 day post-LPS (Figure 6B). See Figure 6C for a complete summary of our post hoc analysis results. These data indicate that genetic ablation of IL-10 has selective influences on the kinetics of morphological responses to TLR-4 stimulation. Furthermore, the *IL-10*^−/−^ responses appeared to show a more complete recovery to the pre-stimulus (*t* = 0) levels in comparison to the findings for the WT microglia for fs2 and fs3 (Figure 6B).

**Figure 7:**
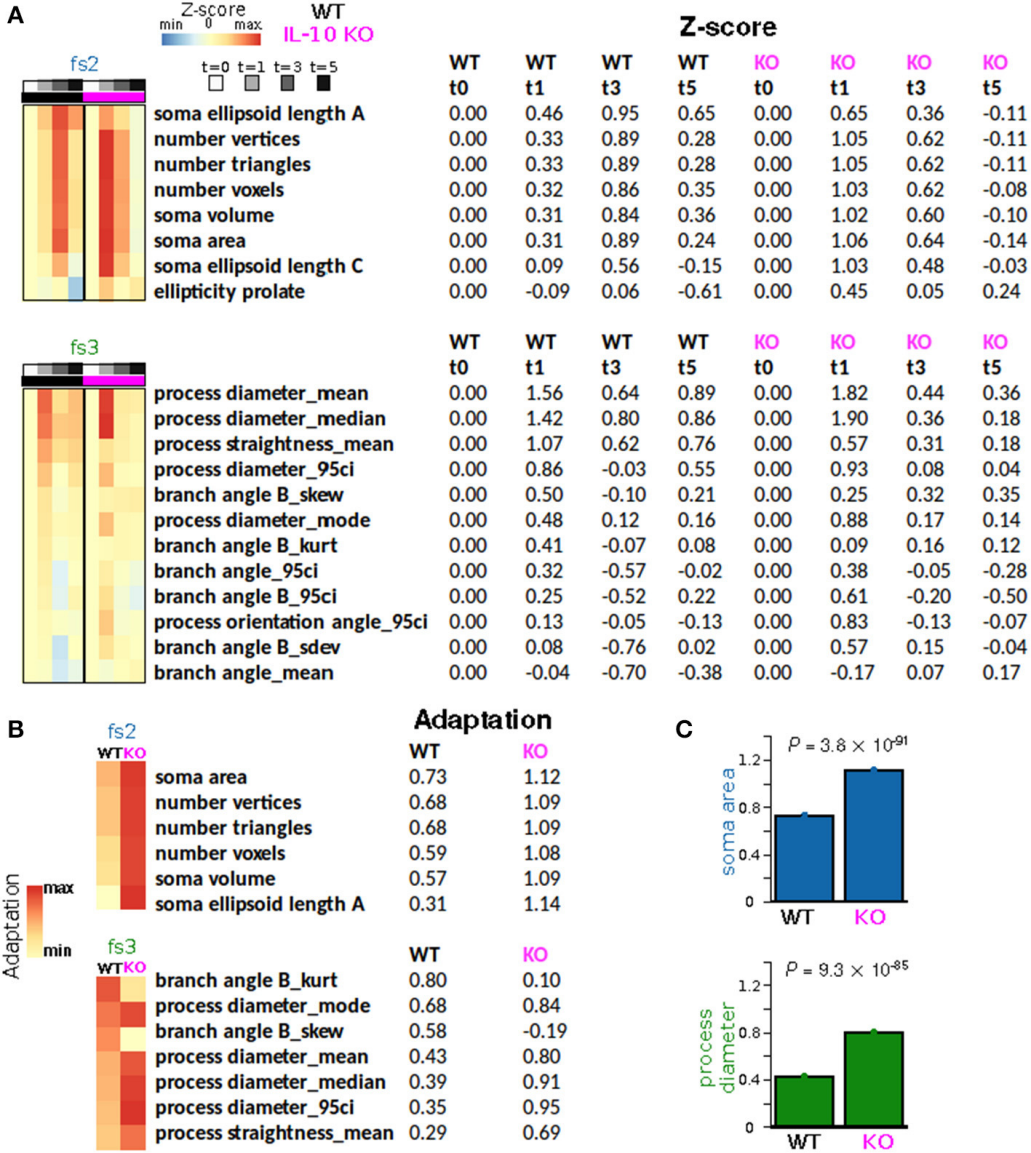
IL-10 restrains morphological adaptation. **(A)** Average Z-scores represented as a function of time for individual features from feature sets fs2 (soma size/shape) and fs3 (process shape). Rows (features) were sorted according to the peak WT average Z-scores. **(B)** Adaptation indices were computed and displayed for a subset of features that met certain criteria (Methods). *IL-10*^−/−^ microglia exhibit enhanced adaptation of key features associated with the shape of somata and processes. **(C)** Specific examples of adaptation indices illustrate the enhancement of morphological adaptation associated with *IL-10*^−/−^. The data were analyzed using two-tailed *t*-test with corrected *p*-values using the Bengamini-Hoshberg procedure.

To quantify morphological adaptation between WT and IL-10 KO, we computed adaptation indices and estimated the standard errors corresponding to these adaptation indices (Figures 7A,B; see Methods). For morphological features that met our criteria for adaptation analysis, all fs2 features showed greater adaptation for the *IL-10* KO genotype, and 5/7 fs3 features showed greater adaptation for *IL-10* KO (Figures 7B,C). When we considered all morphological features, the majority showed significantly greater adaptation for *IL-10* KO (all *P* < 1.4 × 10^−15^, Figure 7B; the *p*-values were computed as described in the Methods section “Morphological adaptation analysis” based on the t-distribution corresponding to an estimate of the T-statistic). Strikingly, these data indicate that the adaptive recovery of morphological features and cytokine responses to infiammatory insult are enhanced by the absence of IL-10 in the cytokine regulatory network.

### *In silico* modeling analysis of IL-10 feedback regulation of TNFα adaptation

**Figure 8:**
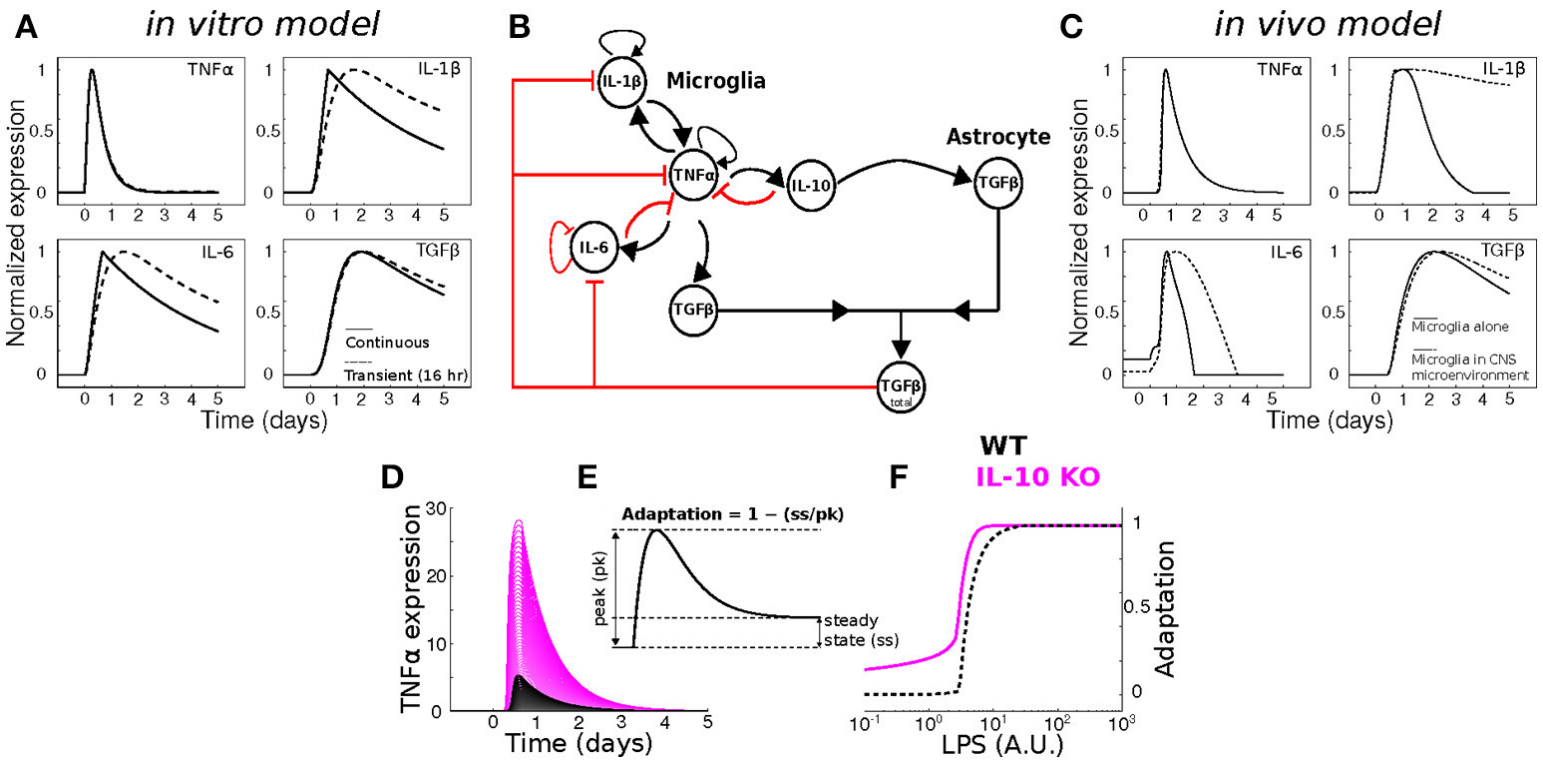
Dynamical modeling analyses of cytokine network regulation. **(A)** Simulation of our *in vitro* cytokine model cannot account for *in vivo* dynamics under conditions of either continuous (dashed) or transient (solid) LPS stimulation. **(B)** Literature-based data-driven cytokine interaction network based on *in vivo* data. **(C)** *In silico* model simulations of *in vivo* cytokine network dynamics (see Methods and Discussion for further explanation of this model). **(D)** Simulations of wildtype (WT; black) and IL-10 KO; magenta) model TNFα response to a range of LPS stimulus magnitudes (arbitrary units). **(E)** Analytic framework for computing adaptive recovery of the TNFα response to LPS. **(F)** LPS concentration response profiles for adaptation of WT and IL-10 KO phenotypes *in silico* show that the IL-10 KO enhances adaptation to LPS (left-shifted curve).

To assess whether our previous model of *in vitro* microglial cytokine dynamics (Anderson et al., 2015) agreed with our *in vivo* cytokine expression measurements, we compared the model simulations (Figure 8A) to our data (Figure 1). Our original model was designed to simulate the microglial response to a continuous LPS stimulus (Figure 8A, dashed traces). To match our *in vivo* experiment, we re-simulated the model with a transient stimulus (Figure 8A, solid traces). Regardless of the LPS stimulus duration, our *in vitro* model did not capture the expression dynamics observed *in vivo* (note that we show data for a 16 h stimulus). In particular, the pronounced degree of adaptation observed for *Il1b* and *Il6 in vivo* suggested that additional negative feedback constraints may enforce adaptive recovery to infection or insult in the CNS.

To quantitatively evaluate network influences on cytokine expression dynamics *in vivo*, we developed a new multi-cellular computational model in which IL-10 from microglia stimulates TGFβ up-regulation by in the surrounding microenvironment (most likely derived from astrocytes) and TGFβ inhibits microglial IL-1β and IL-6 expression (Norden et al., 2014) (Figure 8B). The inclusion of microglia interactions with the CNS environment was critical to account for the *in vivo* cytokine expression dynamics in the simulations (Figure 8C; compare with Figure 1). Simulation of our new neuroinflammation model yielded temporal patterns of cytokine expression that were in qualitative agreement with our experimental data (Figure 1). To examine the implications of negative feedback on the cytokine network, we simulated the effects of *IL-10* KO on the TNFα response to LPS by removing the IL-10 node from the network (Anderson et al., 2015). As expected, removal of IL-10 resulted in enhanced TNFα responses to LPS over a range of doses (Figure 8D). This model prediction is consistent with a wealth of data demonstrating that IL-10 provides feedback inhibition on TNFα expression in glia (Sheng et al., 1995). In agreement with our previous *in vitro* results (Anderson et al., 2015), simulating IL-10 KO in our new model enhanced adaptive recovery of TNFα response to LPS, particularly at lower stimulus intensities (Figures 8E,F). Thus, our simulations and analysis derived from the present *in vivo* data supported the counter-intuitive prediction that IL-10, a feedback inhibitor of TNFα expression, impedes the recovery of TNFα expression toward the baseline level following an LPS stimulus.

## Discussion

This work presents a new approach to investigate microglia by integrating measurements of inflammatory cytokines with novel, unbiased, multivariate analyses to assess microglial morphology and dynamics *in vivo*. Our analyses detected subtle changes in microglial morphology that are missed by conventional qualitative analysis. We also quantitatively characterized adaptation processes based on negative feedback regulation in a novel computational model of CNS inflammation.

It is assumed that microglial morphology affects cell function, and assessments of microglial morphology as a readout of the cell's activation state have been employed for many years (Streit et al., 1999, 2014). Stratifying microglia into classes along a continuum of ramified/hyper-ramified/reactive/ameboid states serves as a useful descriptor of gross changes in morphology. However, such changes (ramified to amoeboid) are rarely seen outside of overt injury sites, such as in traumatic/ischemic lesions. Furthermore, assessment of microglial cell morphology in such lesions has been confounded by the presence of infiltrating monocyte derived macrophages (Mawhinney et al., 2012; Greenhalgh and David, 2014; Bennett et al., 2016; Greenhalgh et al., 2016). As microglial functions are now implicated in the onset of Alzheimer's disease, schizophrenia, epilepsy and intellectual disability (Frick et al., 2013; Abiega et al., 2016; Hong et al., 2016), appropriate assessment of subtle changes in microglial morphology may illuminate their homeostatic and pathophysiological functions. Our current approach circumvents the shortcomings associated with conventionally applied qualitative morphology analyses. For example, when sample images of reconstructed microglia most representative of each cluster were examined, the six clusters were not easily or unambiguously identifiable by visual inspection. Many studies have used 3-D reconstruction software, such as IMARIS, to advance the study of microglia by providing quantitative measures of their morphology (Madore et al., 2013; Erny et al., 2015; Lewitus Gil et al., 2016). Our results show that over one-hundred measurable parameters can be quantified using such analyses. The caveat with these large data sets is that it is difficult to identify significantly different morphological features while controlling for the type I error rate. We have attempted to avoid such errors by incorporating the entire dataset and using appropriate statistical measures such as the optimal discovery procedure (ODP) (Storey et al., 2005, 2007) instead of ANOVA, which is sub-optimal for analyzing temporal dynamics across many features (Storey et al., 2005). Furthermore, for the first time in the assessment of microglial morphology, we employed non-negative matrix factorization (NMF), which is a highly effective technique to define groups of features that are associated with varying degrees of representation in distinct clusters of cells (Lee and Seung, 1999). Spatiotemporal monitoring of microglia morphology in relation to brain injury has been investigated (Morrison and Filosa, 2013) and hierarchical cluster analysis of the morphometric features was used to objectively describe and classify morphological changes under homeostatic and neuropathological conditions (Yamada and Jinno, 2013; Diniz et al., 2016). Our present data build on these hierarchical clustering techniques and provide an approach that is unbiased, sensitive, and can be widely used to study subtle changes in microglial morphology.

After predicting and validating the counter-intuitive notion that IL-10 limits the recovery of TNFα *in vivo*, we sought to investigate whether certain aspects of microglial morphology were also regulated similarly. Using the sensitive, unbiased approaches to assess morphology, we found that adaptation of feature sets associated with soma size/shape (fs2) and process shape (fs3) showed significantly increased adaptation in the absence of IL-10. These findings indicate that IL-10 impedes the recovery of these features of microglial morphology to homeostatic levels in a similar time-course as IL-10 impedes the return of TNFα to baseline. We also considered groups of individual morphological features, the majority of which showed significantly greater adaptation in *IL-10*^−/−^ mice, especially those associated with larger soma size, consistent with microglial activation following LPS application (Kozlowski and Weimer, 2012). Interestingly, features associated with the complexity of branch shape, including features related to the angularity of branching, also showed significantly greater adaptation, consistent with the notion that ramified microglia represent a surveying, non-activated state (Karperien et al., 2013).

We developed a novel computational model to account for the dynamics of cytokine interactions between microglia and the CNS environment based on the *in vivo* cytokine data collected in the current study. In the present two-compartment model, we improved our previously established model of cytokine dynamics which represented a single compartment of microglial cells, regulated through autocrine/paracrine signaling, based on data obtained from primary microglial cell cultures (Anderson et al., 2015). To incorporate the interactions of microglial cells with their *in vivo* microenvironment, we added a second compartment to the model. This compartment was proposed to be dominated by astrocyte-mediated feedback influences on microglia based on astrocyte TGFβ release and consequent feedback inhibition of microglial IL-1β and IL-6 (Norden et al., 2014). CNS neuroinflammation involves an expansive repertoire of cytokines secreted by cell types including neurons, endothelial cells, pericytes, astrocytes, and microglia. Our simplified modeling approach was driven by (a) the dearth of data regarding the cellular sources, temporal expression dynamics, and relative functional influences of the multitude of cytokines, and (b) the motivation to develop a parsimonious model that could capture the complex dynamics of multivariate cytokine expression—as regulated by feedback control of microglial inflammation—without the unnecessary inclusion of unknown molecular interaction parameters. Such simplified models are often considered to be desirable from the perspective of model analysis because model simplification facilitates the identification of critical regulators of the system's function (Huang et al., 2010; Transtrum and Qiu, 2014). While we have not undertaken focused experimental analyses to verify the proposed components of astrocyte-mediated feedback of microglia via TGFβ, our data are consistent with our model analyses. Even if astrocyte-mediated feedback regulation of microglial inflammation were not exclusively controlled by TGFβ, our model illustrates a plausible mechanism from the perspective of the observed process dynamics. Furthermore, we have argued that computational modeling can be utilized for numerous purposes other than accurately recapitulating or predicting the precise mechanisms of function (Anderson and Vadigepalli, in press). Alternatively, as in our study, modeling can be used to show that negative feedback processes—regardless of their precise molecular mechanisms—are sufficient to explain the effect of IL-10 occlusion on the adaptive response to CNS insult. Our model of *in vivo* neuroinflammation captures the dynamics of two glial cell types with distinct regulatory interactions. Based on model simulations, we predicted that the anti-inflammatory cytokine IL-10 would impede the adaptation of TNFα. Our experimental data confirmed this prediction, highlighting the utility of mathematically modeling of complex cytokine networks to investigate neuroinflammation.

Morphological analyses were performed on microglial cells of the spinal cord. This is a particularly relevant region of the CNS to study neuroinflammatory mechanisms, as understanding its effect on neuronal degeneration and regeneration are key to recovery in spinal cord injury. An interesting avenue for further investigation will be to understand if there are region-specific responses during neuroinflammation. It has been demonstrated that there is microglial diversity between different brain regions (Grabert et al., 2016). This is more pronounced in the healthy brain as, in perturbed CNS states, such as aging and pathological conditions, microglial diversity and the homeostatic signature of microglia is lost (Grabert et al., 2016; Keren-Shaul et al., 2017).

Transcriptional analyses of microglia in their various states of activation have contributed greatly to our understanding of these cells (Butovsky et al., 2014; Healy et al., in press). As microglia morphology also reflects their function (Walker et al., 2014), the coupling of morphological data-sets, such as in the present study, with transcriptional profiles of these individual cells, could provide a tool kit to the exact functions of distinct morphologies.

Future extensions of our study include identifying disease or injury-specific dynamic profiles of cytokine networks and correlations with changes in particular aspects of microglial morphology that can reveal functional states, and lead to the identification of molecular mechanisms underlying the coupling between cytokine regulation and microglial morphology. Our results depict a hitherto unrecognized association between the dynamics of cytokine gene expression and microglial morphology in the CNS, and the counter-intuitive role of IL-10 in regulating these dynamics *in vivo*. The combination of mathematical modeling of cytokine networks, *in vivo* validation, and sensitive morphological analysis will be a valuable tool to study changes in microglial responses in CNS injury and disease.

## Author contributions

WA and AG designed experiments, analyzed data, and wrote manuscript. AG performed experiments and WA performed statistical analysis and mathematical modeling. AT performed experiments. SD and RV supervised studies and wrote manuscript.

### Conflict of interest statement

The authors declare that the research was conducted in the absence of any commercial or financial relationships that could be construed as a potential conflict of interest.

## Supplementary material

Supplementary Figure 1 TNFα expression increases to the same extent in both brain and spinal cord tissue after systemic LPS (0.33 mg/kg i.p) at 24 h (*n* = 3–4 mice per group).

## References

[B1] Abiega O. Beccari S. Diaz-Aparicio I. Nadjar A. Layé S. Leyrolle Q. (2016). Neuronal hyperactivity disturbs ATP microgradients, impairs microglial motility, and reduces phagocytic receptor expression triggering apoptosis/microglial phagocytosis uncoupling. PLoS Biol. 14:e1002466 10.1371/journal.pbio.1002466 27228556 PMC4881984

[B2] Anderson W. D. DeCicco D. Schwaber J. S. Vadigepalli R. (2017). A data-driven modeling approach to identify disease-specific multi-organ networks driving physiological dysregulation. PLoS Comput. Biol. 13:e1005627. 10.1371/journal.pcbi.1005627 28732007 PMC5521738

[B3] Anderson W. D. Makadia H. K. Greenhalgh A. D. Schwaber J. S. David S. Vadigepalli R. (2015). Computational modeling of cytokine signaling in microglia. Mol. Biosyst. 11, 3332–3346. 10.1039/C5MB00488H 26440115 PMC5520540

[B4] Anderson W. D. Makadia H. K. Vadigepalli R. (2016). Molecular variability elicits a tunable switch with discrete neuromodulatory response phenotypes. J. Comput. Neurosci. 40, 65–82. 10.1007/s10827-015-0584-2 26621106 PMC4867553

[B5] Anderson W. D. Vadigepalli R. (in press). Modeling cytokine regulatory network dynamics driving neuroinflammation in central nervous system disorders. Drug Dis. Today. 10.1016/j.ddmod.2017.01.003 PMC5609716 28947907

[B6] Badea L. (2008). Extracting gene expression profiles common to colon and pancreatic adenocarcinoma using simultaneous nonnegative matrix factorization, in Pacific Symposium on Biocomputing (Bucharest: Citeseer), 279–290. 18229692

[B7] Bennett M. L. Bennett F. C. Liddelow S. A. Ajami B. Zamanian J. L. Fernhoff N. B.. (2016). New tools for studying microglia in the mouse and human CNS. Proc. Natl. Acad. Sci. U.S.A. 113, E1738–E1746. 10.1073/pnas.1525528113 26884166 PMC4812770

[B8] Benveniste E. N. (1992). Inflammatory cytokines within the central nervous system: sources, function, and mechanism of action. Am. J. Physiol. Cell Physiol. 263, C1–C16. 1636671 10.1152/ajpcell.1992.263.1.C1

[B9] Boutsidis C. Gallopoulos E. (2008). SVD based initialization: a head start for nonnegative matrix factorization. Pattern Recognit. 41, 1350–1362. 10.1016/j.patcog.2007.09.010

[B10] Brudecki L. Ferguson D. A. McCall C. E. El Gazzar M. (2013). MicroRNA-146a and RBM4 form a negative feed-forward loop that disrupts cytokine mRNA translation following TLR4 responses in human THP-1 monocytes. Immunol. Cell Biol. 91, 532–540. 10.1038/icb.2013.37 23897118 PMC3770753

[B11] Brunet J.-P. Tamayo P. Golub T. R. Mesirov J. P. (2004). Metagenes and molecular pattern discovery using matrix factorization. Proc. Natl. Acad. Sci. U.S.A. 101, 4164–4169. 10.1073/pnas.0308531101 15016911 PMC384712

[B12] Butovsky O. Jedrychowski M. P. Moore C. S. Cialic R. Lanser A. J. Gabriely G.. (2014). Identification of a unique TGF-β-dependent molecular and functional signature in microglia. Nat. Neurosci. 17, 131–143. 10.1038/nn.3599 24316888 PMC4066672

[B13] Chakrabarty P. Li A. Ceballos-Diaz C. Eddy James A. Funk Cory C. Moore B.. (2015). IL-10 Alters immunoproteostasis in APP mice, increasing plaque burden and worsening cognitive behavior. Neuron 85, 519–533. 10.1016/j.neuron.2014.11.020 25619653 PMC4320003

[B14] Cieślik M. Bekiranov S. (2014). Combinatorial epigenetic patterns as quantitative predictors of chromatin biology. BMC Genomics 15:76. 10.1186/1471-2164-15-76 24472558 PMC3922690

[B15] Codarri L. Fontana A. Becher B. (2010). Cytokine networks in multiple sclerosis, lost in translation. Curr. Opin. Neurol. 23, 205–211. 10.1097/WCO.0b013e3283391feb 20442570

[B16] Crotti A. Ransohoff R. M. (2016). Microglial physiology and pathophysiology: insights from genome-wide transcriptional profiling. Immunity 44, 505–515. 10.1016/j.immuni.2016.02.013 26982357

[B17] Dabney A. Storey J. D. Warnes G. (2010). qvalue: Q-Value Estimation for False Discovery Rate Control. R package version 1.

[B18] Davalos D. Grutzendler J. Yang G. Kim J. Zuo Y. Jung S. (2005). ATP mediates rapid microglial response to local brain injury *in vivo*. Nat. Neurosci. 6, 752–758. 10.1038/nn1472 15895084

[B19] David S. Greenhalgh A. D. Kroner A. (2015). Macrophage and microglial plasticity in the injured spinal cord. Neuroscience 307, 311–318. 10.1016/j.neuroscience.2015.08.064 26342747

[B20] Diniz D. G. Silva G. O. Naves T. B. Fernandes T. N. Araújo S. C. Diniz J. A. P. (2016). Hierarchical cluster analysis of three-dimensional reconstructions of unbiased sampled microglia shows not continuous morphological changes from stage 1 to 2 after multiple dengue infections in callithrix penicillata. Front. Neuroanat. 10:23 10.3389/fnana.2016.00023 27047345 PMC4801861

[B21] DiSabato D. J. Quan N. Godbout J. P. (2016). Neuroinflammation: the devil is in the details. J. Neurochem. 139, 136–153. 10.1111/jnc.13607 26990767 PMC5025335

[B22] Erny D. Hrabe de Angelis A. L. Jaitin D. Wieghofer P. Staszewski O. David E.. (2015). Host microbiota constantly control maturation and function of microglia in the CNS. Nat. Neurosci. 18, 965–977. 10.1038/nn.4030 26030851 PMC5528863

[B23] Fenn A. M. Henry C. J. Huang Y. Dugan A. Godbout J. P. (2012). Lipopolysaccharide-induced interleukin (IL)-4 receptor-α expression and corresponding sensitivity to the M2 promoting effects of IL-4 are impaired in microglia of aged mice. Brain Behav. Immun. 26, 766–777. 10.1016/j.bbi.2011.10.003 22024136 PMC3288757

[B24] Frick L. R. Williams K. Pittenger C. (2013). Microglial dysregulation in psychiatric disease. Clin. Dev. Immunol. 2013, 10. 10.1155/2013/608654 23690824 PMC3652125

[B25] Gadani S. P. Walsh J. T. Lukens J. R. Kipnis J. (2015). Dealing with danger in the CNS: the response of the immune system to injury. Neuron 87, 47–62. 10.1016/j.neuron.2015.05.019 26139369 PMC4491143

[B26] Gao Y. Church G. (2005). Improving molecular cancer class discovery through sparse non-negative matrix factorization. Bioinformatics 21, 3970–3975. 10.1093/bioinformatics/bti653 16244221

[B27] Gaujoux R. Seoighe C. (2010). A flexible R package for nonnegative matrix factorization. BMC Bioinformatics 11:367. 10.1186/1471-2105-11-367 20598126 PMC2912887

[B28] Grabert K. Michoel T. Karavolos M. H. Clohisey S. Baillie J. K. Stevens M. P.. (2016). Microglial brain region-dependent diversity and selective regional sensitivities to aging. Nat. Neurosci. 19, 504–516. 10.1038/nn.4222 26780511 PMC4768346

[B29] Greenhalgh A. D. David S. (2014). Differences in the phagocytic response of microglia and peripheral macrophages after spinal cord injury and its effects on cell death. J. Neurosci. 34, 6316–6322. 10.1523/JNEUROSCI.4912-13.2014 24790202 PMC6608120

[B30] Greenhalgh A. D. Passos dos Santos R. Zarruk J. G. Salmon C. K. Kroner A. David S. (2016). Arginase-1 is expressed exclusively by infiltrating myeloid cells in CNS injury and disease. Brain Behav. Immun. 56, 61–67. 10.1016/j.bbi.2016.04.013 27126514

[B31] Guillot-Sestier M.-V. Doty Kevin R. Gate D. Rodriguez J. Jr. Leung Brian P. Rezai-Zadeh K.. (2015). Il10 Deficiency rebalances innate immunity to mitigate alzheimer-like pathology. Neuron 85, 534–548. 10.1016/j.neuron.2014.12.068 25619654 PMC4352138

[B32] Healy L. M. Perron G. Won S.-Y. Rao V. T. S. Guiot M.-C. Moore C.. (in press). Differential transcriptional response profiles in human myeloid cell populations. Clin. Immunol. 10.1016/j.clim.2016.04.006 27094466

[B33] Henry C. J. Huang Y. Wynne A. M. Godbout J. P. (2009). Peripheral Lipopolysaccharide (LPS) challenge promotes microglial hyperactivity in aged mice that is associated with exaggerated induction of both pro-inflammatory IL-1β and anti-inflammatory IL-10 cytokines. Brain Behav. Immun. 23, 309–317. 10.1016/j.bbi.2008.09.002 18814846 PMC2692986

[B34] Hines D. J. Hines R. M. Mulligan S. J. Macvicar B. A. (2009). Microglia processes block the spread of damage in the brain and require functional chloride channels. Glia 57, 1610–1618. 10.1002/glia.20874 19382211

[B35] Hong S. Beja-Glasser V. F. Nfonoyim B. M. Frouin A. Li S. Ramakrishnan S.. (2016). Complement and microglia mediate early synapse loss in Alzheimer mouse models. Science 352, 712–716. 10.1126/science.aad8373 27033548 PMC5094372

[B36] Huang Z. Chu Y. Hahn J. (2010). Model simplification procedure for signal transduction pathway models: an application to IL-6 signaling. Chem. Eng. Sci. 65, 1964–1975. 10.1016/j.ces.2009.11.035

[B37] Ishii H. Tanabe S. Ueno M. Kubo T. Kayama H. Serada S.. (2013). ifn-γ-dependent secretion of IL-10 from Th1 cells and microglia/macrophages contributes to functional recovery after spinal cord injury. Cell Death Dis. 4:e710. 10.1038/cddis.2013.234 23828573 PMC3730408

[B38] Kampstra P. (2008). Beanplot: a boxplot alternative for visual comparison of distributions. J. Stat. Softw. 28, 1–9. 10.18637/jss.v028.c0127774042

[B39] Karperien A. Ahammer H. Jelinek H. F. (2013). Quantitating the subtleties of microglial morphology with fractal analysis. Front. Cell. Neurosci. 7:3. 10.3389/fncel.2013.00003 23386810 PMC3558688

[B40] Keren-Shaul H. Spinrad A. Weiner A. Matcovitch-Natan O. Dvir-Szternfeld R. Ulland T. K.. (2017). A unique microglia type associated with restricting development of Alzheimer's disease. Cell 169, 1276–1290. 10.1016/j.cell.2017.05.018 28602351

[B41] Kettenmann H. Kirchhoff F. Verkhratsky A. (2013). Microglia: new roles for the synaptic stripper. Neuron 77, 10–18. 10.1016/j.neuron.2012.12.023 23312512

[B42] Kilkenny C. Browne W. J. Cuthill I. C. Emerson M. Altman D. G. (2010). Improving bioscience research reporting: the ARRIVE guidelines for reporting animal research. PLoS Biol. 8:e1000412. 10.1371/journal.pbio.1000412 20613859 PMC2893951

[B43] Kongsui R. Beynon S. B. Johnson S. J. Walker F. R. (2014). Quantitative assessment of microglial morphology and density reveals remarkable consistency in the distribution and morphology of cells within the healthy prefrontal cortex of the rat. J. Neuroinflammation 11:182 10.1186/s12974-014-0182-7 25343964 PMC4213482

[B44] Kozlowski C. Weimer R. M. (2012). An automated method to quantify microglia morphology and application to monitor activation state longitudinally *in vivo*. PLoS ONE 7:e31814. 10.1371/journal.pone.0031814 22457705 PMC3294422

[B45] Kroner A. Greenhalgh Andrew D. Zarruk Juan G. Passos dos Santos R. Gaestel M. David S. (2014). TNF and increased intracellular iron alter macrophage polarization to a detrimental M1 phenotype in the injured spinal cord. Neuron 83, 1098–1116. 10.1016/j.neuron.2014.07.027 25132469

[B46] Lee D. D. Seung H. S. (1999). Learning the parts of objects by non-negative matrix factorization. Nature 401, 788–791. 10.1038/44565 10548103

[B47] Lee D. D. Seung H. S. (2001). Algorithms for non-negative matrix factorization, in Advances in Neural Information Processing Systems (Cambridge: The MIT Press), 556–562.

[B48] Lewitus Gil M. Konefal Sarah C. Greenhalgh Andrew D. Pribiag H. Augereau K. Stellwagen D. (2016). Microglial TNF-α suppresses cocaine-induced plasticity and behavioral sensitization. Neuron 90, 483–491. 10.1016/j.neuron.2016.03.030 27112496 PMC4860141

[B49] Liu T. Wang Q.-G. Huang H.-P. (2013). A tutorial review on process identification from step or relay feedback test. J. Process Control 23, 1597–1623. 10.1016/j.jprocont.2013.08.00321777915

[B50] Madore C. Joffre C. Delpech J. C. De Smedt-Peyrusse V. Aubert A. Coste L.. (2013). Early morphofunctional plasticity of microglia in response to acute lipopolysaccharide. Brain Behav. Immun. 34, 151–158. 10.1016/j.bbi.2013.08.008 23994463

[B51] Madsen P. M. Motti D. Karmally S. Szymkowski D. E. Lambertsen K. L. Bethea J. R.. (2016). Oligodendroglial TNFR2 mediates membrane TNF-dependent repair in experimental autoimmune encephalomyelitis by promoting oligodendrocyte differentiation and remyelination. J. Neurosci. 36, 5128–5143. 10.1523/JNEUROSCI.0211-16.2016 27147664 PMC4854972

[B52] Marchini J. L. Heaton C. Ripley B. D. (2013). Fastica: Fastica Algorithms to Perform ICA and Projection Pursuit, R Package Version 1. Oxford: Oxford University Press Available online at: http://cran.r-project.org/web/packages/fastICA/index.html

[B53] Mawhinney L. A. Thawer S. G. Lu W.-Y. Rooijen N. V. Weaver L. C. Brown A.. (2012). Differential detection and distribution of microglial and hematogenous macrophage populations in the injured spinal cord of lys-EGFP-ki transgenic mice. J. Neuropathol. Exp. Neurol. 71, 180–197. 10.1097/NEN.0b013e3182479b41 22318123

[B54] Montefusco F. Akman O. E. Soyer O. S. Bates D. G. (2016). Ultrasensitive negative feedback control: a natural approach for the design of synthetic controllers. PLoS ONE 11:e0161605. 10.1371/journal.pone.0161605 27537373 PMC5004582

[B55] Montgomery S. L. Narrow W. C. Mastrangelo M. A. Olschowka J. A. O'Banion M. K. Bowers W. J. (2013). Chronic neuron- and age-selective down-regulation of TNF receptor expression in triple-transgenic alzheimer disease mice leads to significant modulation of amyloid- and tau-related pathologies. Am. J. Pathol. 182, 2285–2297. 10.1016/j.ajpath.2013.02.030 23567638 PMC3668024

[B56] Morrison H. Filosa J. (2013). A quantitative spatiotemporal analysis of microglia morphology during ischemic stroke and reperfusion. J. Neuroinflammation 10:4 10.1186/1742-2094-10-4 23311642 PMC3570327

[B57] Norden D. M. Fenn A. M. Dugan A. Godbout J. P. (2014). TGFβ produced by IL-10 redirected astrocytes attenuates microglial activation. Glia 62, 881–895. 10.1002/glia.22647 24616125 PMC4061706

[B58] Ogunnaike B. A. (2011). Random Phenomena: Fundamentals of Probability and Statistics for Engineers. Boca Raton, FL CRC Press.

[B59] Ogunnaike B. A. Ray W. H. (1994). Process Dynamics, Modeling, and Control. New York, NY: Oxford University Press.

[B60] Park J. Brureau A. Kernan K. Starks A. Gulati S. Ogunnaike B.. (2014). Inputs drive cell phenotype variability. Genome Res. 24, 930–941. 10.1101/gr.161802.113 24671852 PMC4032857

[B61] R-Core-Team (2016) R: A Language and Environment for Statistical Computing. Vienna: R Foundation for Statistical Computing.

[B62] Schafer D. P. Lehrman E. K. Kautzman A. G. Koyama R. Mardinly A. R. Yamasaki R.. (2012). Microglia sculpt postnatal neural circuits in an activity and complement-dependent manner. Neuron 74, 691–705. 10.1016/j.neuron.2012.03.026 22632727 PMC3528177

[B63] Schafer D. P. Stevens B. (2015). Microglia function in central nervous system development and plasticity. Cold Spring Harb. Perspect. Biol. 7:a020545. 10.1101/cshperspect.a020545 26187728 PMC4588063

[B64] Schmittgen T. D. Livak K. J. (2008). Analyzing real-time PCR data by the comparative C(T) method. Nat. Protoc. 3, 1101–1108. 10.1038/nprot.2008.73 18546601

[B65] Sheng W. S. Hu S. Kravitz F. H. Peterson P. K. Chao C. C. (1995). Tumor necrosis factor alpha upregulates human microglial cell production of interleukin-10 *in vitro*. Clin. Diagn. Lab. Immunol. 2, 604–608. 8548541 10.1128/cdli.2.5.604-608.1995 PMC170206

[B66] Sierra A. Abiega O. Shahraz A. Neumann H. (2013). Janus-faced microglia: beneficial and detrimental consequences of microglial phagocytosis. Front. Cell. Neurosci. 7:6. 10.3389/fncel.2013.00006 23386811 PMC3558702

[B67] Sipe G. O. Lowery R. L. Tremblay M. È. Kelly E. A. Lamantia C. E. Majewska A. K. (2016). Microglial P2Y12 is necessary for synaptic plasticity in mouse visual cortex. Nat. Commun. 7:10905. 10.1038/ncomms10905 26948129 PMC4786684

[B68] Siqueira Mietto B. Kroner A. Girolami E. I. Santos-Nogueira E. Zhang J. David S. (2015). Role of IL-10 in resolution of inflammation and functional recovery after peripheral nerve injury. J. Neurosci. 35, 16431–16442. 10.1523/JNEUROSCI.2119-15.2015 26674868 PMC6605511

[B69] Šišková Z. Tremblay M-. È. (2013). Microglia and synapse: interactions in health and neurodegeneration. Neural Plast. 2013:425845 10.1155/2013/425845 24392228 PMC3874338

[B70] Storey J. D. Dai J. Y. Leek J. T. (2007). The optimal discovery procedure for large-scale significance testing, with applications to comparative microarray experiments. Biostatistics 8, 414–432. 10.1093/biostatistics/kxl019 16928955

[B71] Storey J. D. Xiao W. Leek J. T. Tompkins R. G. Davis R. W. (2005). Significance analysis of time course microarray experiments. Proc. Natl. Acad. Sci. U.S.A. 102, 12837–12842. 10.1073/pnas.0504609102 16141318 PMC1201697

[B72] Storey J. Leek J. Bass A. (2015). edge: Extraction of Differential Gene Expression. R package version 2.6.0. 16357033

[B73] Streit W. J. Walter S. A. Pennell N. A. (1999). Reactive microgliosis. Prog. Neurobiol. 57, 563–581. 10.1016/S0301-0082(98)00069-0 10221782

[B74] Streit W. J. Xue Q.-S. Tischer J. Bechmann I. (2014). Microglial pathology. Acta Neuropathol. Commun. 2:142. 10.1186/s40478-014-0142-6 25257319 PMC4180960

[B75] Transtrum M. K. Qiu P. (2014). Model reduction by manifold boundaries. Phys. Rev. Lett. 113:098701 10.1103/PhysRevLett.113.098701 25216014 PMC4425275

[B76] Tremblay M.-È. Lowery R. L. Majewska A. K. (2010). Microglial interactions with synapses are modulated by visual experience. PLoS Biol. 8:e1000527. 10.1371/journal.pbio.1000527 21072242 PMC2970556

[B77] Vasek M. J. Garber C. Dorsey D. Durrant D. M. Bollman B. P. Soung A.. (2016). A complement–microglial axis drives synapse loss during virus-induced memory impairment. Nature 534, 538–543. 10.1038/nature18283 27337340 PMC5452615

[B78] Walker F. R. Beynon S. B. Jones K. A. Zhao Z. Kongsui R. Cairns M.. (2014). Dynamic structural remodelling of microglia in health and disease: a review of the models, the signals and the mechanisms. Brain Behav. Immun. 37, 1–14. 10.1016/j.bbi.2013.12.010 24412599

[B79] Witcher K. G. Eiferman D. S. Godbout J. P. (2015). Priming the inflammatory pump of the CNS after traumatic brain injury. Trends Neurosci. 38, 609–620. 10.1016/j.tins.2015.08.002 26442695 PMC4617563

[B80] Yamada J. Jinno S. (2013). Novel objective classification of reactive microglia following hypoglossal axotomy using hierarchical cluster analysis. J. Comp. Neurol. 521, 1184–1201. 10.1002/cne.23228 22987820

