# Supplementary figures and images for: Novel Influences of IL-10 on CNS Inflammation Revealed by Integrated Analyses of Cytokine Networks and Microglial Morphology
Source: Front Cell Neurosci. 2017 Aug 14;11:233. doi: 10.3389/fncel.2017.00233 (PMC5557777; doi:10.3389/fncel.2017.00233)

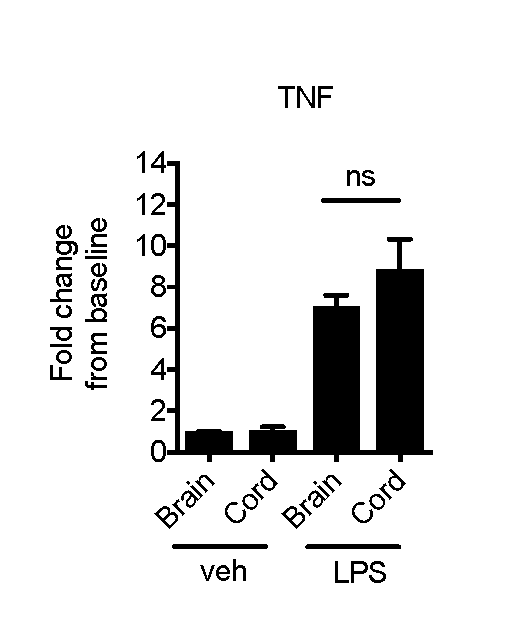

Supplement: Supplementary Figure 1 — TNFα expression increases to the same extent in both brain and spinal cord tissue after systemic LPS (0.33 mg/kg i.p) at 24 h (n = 3–4 mice per group). [file Image1.TIFF]
